# Receptor architecture shapes the regional organization of the macaque cingulate cortex

**DOI:** 10.1007/s00429-026-03186-6

**Published:** 2026-10-09

**Authors:** Lucija Rapan, Camille Giacometti, Meiqi Niu, Nicola Palomero-Gallagher

**Affiliations:** 1 C. & O. Vogt Institute for Brain Research, Heinrich-Heine-University, 40225 Düsseldorf, Germany; 2 Institute of Neuroscience and Medicine (INM-1), Research Centre Jülich, Jülich, Germany

**Keywords:** Cingulate sulcus, Receptor mapping, Midcingulate cortex, Cytoarchitecture, Macaque, 3D parcellation map

## Abstract

The cingulate cortex plays a central role in emotion, motivation, decision-making and motor control, yet its internal organization in the macaque brain remains debated. In the present study, we examined the cyto- and receptor architectonic organization of the macaque cingulate cortex and provide a 3D parcellation of the identified areas in both surface and volumetric representations. Multivariate receptor analyses consistently separated areas of the anterior cingulate cortex (ACC) and anterior midcingulate cortex (aMCC) from those of the posterior midcingulate cortex (pMCC) and posterior cingulate cortex (PCC), supporting the definition of four principal cingulate regions in the macaque brain. These regions are characterized by distinct receptor balances: the ACC is enriched with glutamatergic and monoaminergic receptors; the aMCC exhibits high densities of modulatory receptors; the pMCC is characterized by overall lowest receptor densities; and the PCC displays intermediate receptor densities with relatively higher GABA_A_ and M_3_ receptors. Within the ACC, receptor architecture revealed differentiation of subgenual, gyral and sulcal portions, while the PCC contained subdivisions corresponding to dorsal and ventral PCC. Cytoarchitectonic and receptor analyses further demonstrated that cortex along the dorsal bank of the cingulate sulcus (cgs) contains distinct cingulate areas extending from the sulcal fundus onto the dorsal wall, indicating that it forms part of the cingulate cortex rather than the frontal cortex or a transitional zone between those regions. Together, these findings provide a comprehensive anatomical and neurochemical framework for the macaque cingulate cortex and address the long-standing controversy regarding the dorsal bank of the cgs.

**Supplementary Information:**

The online version contains supplementary material available at https://doi.org/10.1007/s00429-026-03186-6.

## Introduction

The cingulate cortex is a functionally heterogeneous and highly interconnected region that plays a central role in emotion regulation, skeletomotor control, and memory-related processes (Rolls 2019). It serves as a key node within multiple large-scale brain networks (Hutchison et al. 2012), including the default mode network (DMN) (Buckner et al. 2008; Mantini et al. 2011) and the ventral salience network (Touroutoglou et al. 2016), underscoring its integrative role in coordinating internally and externally directed cognition (Amiez and Procyk 2019; Devinsky et al. 1995; Leech et al. 2011). Consequently, structural and neurochemical alterations within cingulate circuits have been increasingly implicated in the onset and progression of numerous psychiatric and neurodegenerative disorders, including depression, anxiety, schizophrenia, autism spectrum disorders, and Alzheimer’s disease (Buckner et al. 2005; Vogt 2009). As one of the closest primate models to the human brain, the macaque provides a valuable framework for investigating conserved organizational principles as well as evolutionary divergences in cytoarchitectonic and receptor architecture (Rapan et al. 2022).

**Fig. 1:**
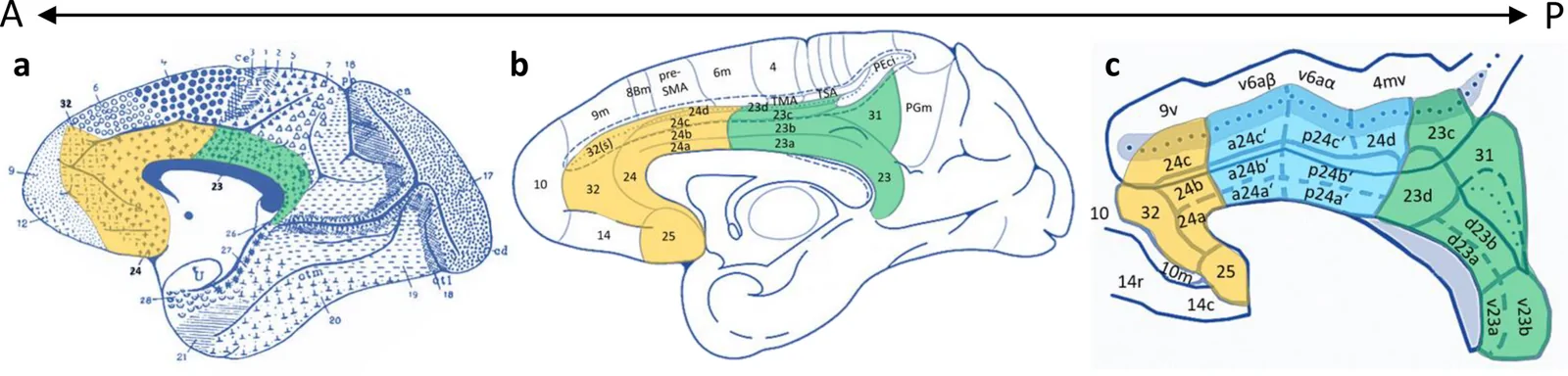
Modified maps of previously published parcellations of the primate cingulate cortex. View of the medial surface of the primate hemisphere depicting maps of cingulate cortex according to **a** Brodmann (1905), **b** Morecraft et al. (2012) and **c** Vogt et al. (2005). Colors code for distinct regions: ACC (orange), MCC (light blue) and PCC (green). See main text for further details. Arrow indicates rostro-caudal orientation. *A* anterior, *P* posterior

Early primate work by Brodmann (Brodmann 1905) (Fig. 1) distinguished anterior (areas 24 and 32) and posterior (area 23) cingulate compartments, primarily based on differences in laminar organization. The anterior division was characterized as agranular or dysgranular, whereas the posterior division exhibited a well-developed granular layer IV. Note that Brodmann’s map of the guenon, which is used as a proxy for the macaque brain, does not contain an area 31. Indeed, Brodmann writes that guenon area 23 must broadly correspond to human areas 23 and 31 (Brodmann 1905). Brodmann also mentioned a transitional region between areas 24 and 23 in the human brain, which he referred to as the midcingulate cortex, although he did not depict it in his parcellation scheme (Brodmann 1909).

Subsequent studies in macaque (Morecraft et al. 2004, 2012; Vogt et al. 2005) further elaborated this rostro–caudal organization, distinguishing rostral areas (25, 32, 24) from caudal areas (23, 31) and introducing additional dorso–ventral subdivisions across the gyrus, sulcal banks, and fundus (Fig. 1). These findings emphasized that the transition from allocortex (e.g., area 33) to neocortex (e.g., areas 24a–d, 23a–d) occurs in a stepwise rather than gradual manner. Although Morecraft et al. (2012) did not recognize the midcingulate region as a distinct compartment, later cyto- and receptor-architectonic studies in both human and macaque established the midcingulate cortex (MCC) as a structurally and functionally distinct region, complementing the anterior (ACC) and posterior (PCC) cingulate cortices in a tripartite organizational model (Palomero-Gallagher et al. 2009; Vogt et al. 1995, 2005). Within the MCC, anterior (aMCC) and posterior (pMCC) subdivisions were identified based on cytoarchitectonic and receptor differences, corresponding in part to functionally defined cingulate motor areas (Dum and Strick 2002). However, while Vogt and Morecraft differed in their subdivision of areas 32 and 23, neither proposed further subdivisions of areas 25 or 31 (Fig. 1).

These differences in parcellation schemes have important implications beyond anatomical description. Inconsistent definitions of cingulate subdivisions limit comparability across studies, complicate translational analyses, and hinder precise identification of homologous regions between macaque and human brains. From a translational neuroscience perspective, variability in macaque cingulate maps affects seed placement in neuroimaging studies and targeting in electrophysiological recordings.

A central point of debate, which strongly impacts translational research, concerns the classification of cortex along the dorsal bank of the cingulate sulcus (cgs). In the human brain, both dorsal and ventral walls of the cgs contain cingulate areas; however, whether a similar arrangement exists in the macaque remains controversial (Dum and Strick 2002; Paxinos et al. 2008; Matelli et al. 1991; Petrides and Pandya 1994.; Vogt et al. 2005). The widely used Paxinos atlas (2008) designates dorsal bank regions as transitional areas (9/32, 8/32, and 6/32). Other authors have interpreted the dorsal bank as belonging to the cingulate cortex, emphasizing similarities to pregenual area 32 (Petrides and Pandya 1994; Morecraft et al. 2012), whereas others have considered it more similar to the ventral bank subdivisions of area 24 (Matelli et al. 1991). In contrast, Vogt et al. (2005) concluded that the dorsal bank does not share defining cytoarchitectonic features with the cingulate cortex and instead corresponds to prefrontal area 9v and adjacent (pre)motor regions (v6aβ, v6aα, and 4mv), thereby restricting cingulate areas primarily to the ventral wall and fundus (Fig. 1).

To address open questions regarding the organization of the macaque cingulate cortex, we performed a detailed re-evaluation of existing maps applying a multimodal and quantitative approach. The ensuing parcellation scheme enables cross-species analyses and facilitates precise identification of homologous cingulate regions in macaque and human brains. We applied a quantitative cytoarchitectonic approach to define statistically significant borders in cell-body silver-stained sections and, in parallel, examined receptor distribution patterns obtained by *in-vitro* autoradiography. Given the heterogeneous distribution of classical neurotransmitter receptors throughout the cortex (Palomero-Gallagher et al. 2008), receptor density analyses provide complementary molecular criteria to cytoarchitecture and can reveal organizational features not captured by laminar characterization alone (Palomero-Gallagher and Zilles 2019). More importantly, the simultaneous analysis of multiple receptors has proven to be a valuable approach when aiming to establish a link between structural and functional segregation of the brain in both human and macaque brains (Niu et al. 2020; Palomero-Gallagher et al. 2009; Rapan et al. 2021, 2022, 2023). While receptor distributions do not directly define cortical function, their density patterns reflect fundamental cortical organization, including excitatory, inhibitory, and modulatory mechanisms underlying memory, learning, arousal, and behavior (Palomero-Gallagher et al. 2015; Rolls 2019; Zilles et al. 2002). Specifically, we measured densities of 14 different receptor types belonging to the classical neurotransmitter systems—glutamatergic (AMPA, kainate, NMDA), GABAergic (GABA_A_, GABA_B_, GABA_A/Bz_), muscarinic (M_1_, M_2_, M_3_), noradrenergic (α_1_, α_2_), serotonergic (5-HT_1A_, 5-HT_2_) and dopaminergic (D_1_).

Thus, the present study aims to clarify key aspects of macaque cingulate organization. Specifically, we address the following questions: (1) can potential subdivisions be recognized within established cingulate areas, such as 25, 23 and 31; (2) do differences in receptor architecture of the identified areas confirm the existence of the ACC, MCC and PCC regions, with their corresponding subregions, i.e., subgenual ACC (sACC)/pregenual ACC (pACC), aMCC/pMCC and dorsal PCC (dPCC)/ventral (vPCC), respectively, as defined by Vogt et al. (2005); (3) does the cortex along the dorsal wall of the macaque cingulate sulcus constitute part of the cingulate cortex rather than be classified as transitional or frontal cortex; and (4) to what extent is the receptor architecture of the macaque cingulate cortex comparable to that reported in the human brain (Palomero-Gallagher et al. 2009).

Finally, we provide a comprehensive 3D map of the macaque cingulate cortex integrated within the MEBRAINS Multilevel Macaque Atlas, serving as a precise reference for the localization of seed regions in *in-vivo* neuroimaging and tracer studies, while simultaneously functioning as a robust translational model for cross-species investigations.

## Material and methods

### Tissue processing

For cytoarchitectonic analysis, both hemispheres of an adult male macaque (*Macaca mulatta*; brain ID DP1; 8 years old; gifted by Professor Deepak N. Pandya) were used. The animal was deeply anesthetized with sodium pentobarbital and transcardially perfused with cold saline followed by 10% buffered formalin. The brain was removed and stored in buffered formalin until further processing.

Prior to the sectioning step, brain ID DP1 was dehydrated and embedded in paraffin in order to preserve soft cortical tissue and serially cut in the coronal plane with a large-scale microtome, resulting in 3305 20-µm-thick whole-brain sections. Every fifth section was mounted on gelatine coated slides. Paraffin was removed and sections were rehydrated by a two-step washing (each of 10 min) with Xem-200 („Xylol-Ersatz-Medium “, Vogel, Diatec Labortechnik GmbH) followed by graded washes in alcohol (10 min each in 100%, 96% and 70% propanol) and finally a rinse in a pure water. To visualize cell bodies, sections were stained with a modified silver method (Merker 1983), which provides a high contrast between cell bodies and neuropil. Sections were pretreated for 4 h in 4% formic acid, followed by overnight incubation in 10% formic acid/30% peroxide. After thorough washing, they were immersed twice for 5 min in 1% acetic acid and developed in freshly prepared physical developer under constant agitation until cell bodies appeared dark grey/black, with progress monitored microscopically. Development was stopped by two 5 min washes in 1% acetic acid. Sections were fixed for 5 min in T-Max fixative (Kodak; 2:7 with distilled water), dehydrated in ascending alcohols (70%, 96%, 100%; 5 min each), cleared in xylene (2 × 5 min), and coverslipped with DePex.

To complement this analysis, three additional adult male macaques (*Macaca fascicularis*; males; brain IDs 11,530, 11,539, 11,543; 7 ± 1 years old; from Covance Laboratories, Münster, Germany), were used primarily for receptor architectonic analysis. These animals were humanely euthanized with intravenous sodium pentobarbital. Brains were immediately extracted with meninges and blood vessels intact to preserve cortical layer I. For receptor autoradiography, only unfixed deep-frozen tissue was used (Palomero-Gallagher and Zilles 2019). Cerebellum and brainstem were removed, and hemispheres were divided into anterior and posterior slabs at the most caudal portion of the central sulcus. Left hemispheres of brains 11,530 and 11,543 and both hemispheres of brain 11,539 were analyzed. Slabs were shock frozen in N-methylbutane at –40°C for 10–15 min, then stored at –80°C until sectioning.

Unfixed frozen slabs were serially sectioned in the coronal plane using a large scale cryostat at − 20 °C, into 20µm-thick sections, which were thaw-mounted on gelatin-coated glass slides. Sections were left to air dry and stored overnight in air-tight plastic bags at − 20°C. Serial sections were used for the visualization of 14 distinct receptors types, i.e., for glutamate (AMPA, kainate, NMDA), gamma-aminobutyric acid (GABA; GABA_A_, GABA_B_, GABA_A_ associated benzodiazepine binding sites [Bz]), acetylcholine (M_1_, M_2_, M_3_), noradrenalin (α_1_, α_2_), serotonin (5-HT_1A_, 5-HT_2_) and dopamine (D_1_), as well as for the visualization of cell bodies (see previous section) using previously published protocols (see Table 1 in Palomero-Gallagher and Zilles 2019). In short, protocols consisted of three subsequent steps: a preincubation, a main incubation and a rinsing step. The preincubation is carried out to rehydrate sections and to remove endogenous ligands that could block the binding sites. During the main incubation, two parallel experiments are conducted to test the specific binding ability of each ligand. In one, sections were incubated in a buffer solution with tritiated ligand to identify total binding of each ligand type. In the second, neighbouring sections were incubated in buffer solution containing the tritiated ligand and a receptor type-specific displacer in a 1000-fold higher concentration to visualize non-specific binding of the same ligand. Finally, the difference between total and non-specific binding demonstrates the specific binding ability for each ligand. In this study, specificity of ligands used resulted in a non-specific binding of less than 5% of the total binding. In the rinsing step, the binding process was stopped, and free ligand and buffer salts removed. Air-dried, radioactive sections were then co-exposed with plastic tritium-standards (calibrated for protein density, and with known increasing concentrations of radioactivity) against β radiation-sensitive films (Hyperfilm®, Amersham) for 4–18 weeks depending on the analyzed ligand (Palomero-Gallagher and Zilles 2019). Animal care complied with NIH Guide for Care and Use of Laboratory Animals, and European Communities Council Directive.

### Identification of cytoarchitectonic borders

Histological sections were scanned using a light microscope (Axioplan 2 imaging, ZEISS, Germany) with a motorized stage controlled by KS400® and Axiovision software (Zeiss, Germany), a 6.3 × 1.25 Planapo® objective, and a CCD camera (Axiocam MRm, ZEISS, Germany) or by means of a TISSUEscope HS (Huron, Canada). Digital images were generated by stitching frames of 524 × 524 µm (512 × 512 pixels; 1 µm/pixel; 8-bit grey scale).

The starting point for the present parcellation was visual inspection to identify the cytoarchitectonic areas identified and described by previously published cytoarchitectonic literature of the macaque cingulate cortex (Morecraft et al. 2012; Palomero-Gallagher et al. 2013; Vogt et al. 2005).

Subsequently, the quantitative cytoarchitectonic analysis was based on use of the grey level index (GLI; Schleicher et al. 2009), where volume density of cell bodies is established relative to neuropil density. GLI images were generated from three neighboring sections per area, and GLI profiles were extracted perpendicularly to the cortical surface between the border of layers I/II and layer VI/white matter (Palomero-Gallagher and Zilles 2019). Each profile was parameterized into ten features describing the laminar distribution of cell body volume fraction, yielding standardized feature vectors for multivariate analyses (Schleicher et al. 2009). Areal borders were identified by quantifying differences between adjacent profiles using Mahalanobis distance (MD; Mahalanobis et al. 1949). Profiles were grouped into sliding blocks, and MD values between adjacent blocks were plotted as distance functions, and the procedure was repeated with increasing number of profiles per block (10–24 profiles) (Schleicher et al. 2009). Significant MD maxima were verified with Hotelling’s T^2^ test and Bonferroni correction. Borders were accepted only if maxima were reproducible across multiple block sizes and confirmed in three consecutive sections, excluding artifacts such as ruptures, folds, vessels, or atypical cell clusters.

### Visualization and quantification of receptor densities

We used quantitative *in-vitro* receptor autoradiography to visualize binding sites of native receptors expressed on the cell membrane. The advantage of this method is that it can be carried out on a large number of large sections, alongside the possibility of precise quantification and a high specificity (Palomero-Gallagher and Zilles 2019).

Autoradiographs were digitized with an image analysis system consisting of a source of homogenous light and a CCD-camera (Axiocam MRm, Zeiss, Germany) with an S-Orthoplanar 60-mm macro lens (Zeiss, Germany) corrected for geometric distortions, connected to the image acquisition and processing system Axiovision (Zeiss, Germany). Spatial resolution of the resulting images was 3000×4000 pixels; 8-bit gray value resolution). The gray values of the digitized autoradiographs code for concentrations of radioactivity. To transform gray values into fmol binding sites/mg protein, a linearization of the digitized autoradiographs had to be performed in a two-steps process, carried out with in house developed Matlab (The MathWorks, Inc. Natrick, MA) scripts. First, the gray value images of the plastic tritium-standards were used to compute the calibration curve, which defines the non-linear relationship between gray values and concentrations of radioactivity. Then radioactivity concentration R was then converted to binding site concentration Cb in fmol/mg protein using following equation:

$$Cb=\frac{R}{E*B*Wb*Sa}*\frac{KD+L}{L}$$

where E is the efficiency of the scintillation counter used to determine the amount of radioactivity in the incubation buffer (depends on the actual counter), B is the number of decays per unit of time and radioactivity (Ci/min), Wb the protein weight of a standard (mg), Sa the specific activity of the ligand (Ci/mmol), KD the dissociation constant of the ligand (nM), and L the free concentration of the ligand during incubation (nM). For details see Palomero-Gallagher and Zilles (2019) and Zilles et al. (2002). For visualization purposes, a linear contrast enhancement and pseudo-color coding of autoradiographs was applied, using a specter of eleven colors with equally spaced density ranges (red color for highest and black for lowest receptor concentration levels) (Palomero-Gallagher and Zilles 2019).

Measurement of receptor binding sites was performed by computing the surface below receptor profiles, which were extracted from the linearized autoradiographs using in house developed scripts for Matlab (The MathWorks, Inc. Natrick, MA) in a manner comparable to the procedure described above for GLI profiles (Palomero-Gallagher and Zilles 2019). However, for receptor profiles the outer contour line was defined following the pial surface, and not the border between layers I and II. Thus, for each of the four hemispheres examined, mean densities (i.e., averaged over all cortical layers) of each of the 14 different receptors in 28 cytoarchitectonically defined areas were calculated. For visualization purposes, both absolute receptor densities (averaged across hemispheres) and normalized receptor densities were displayed as polar coordinate plots (“receptor fingerprints”) using an in-house Python (v3.13) script, highlighting the relative balance of receptor types within each cytoarchitectonically defined area.

### 3D map of the macaque cingulate cortex

To visualize the spatial location and extent of the identified cortical areas within the three-dimensional space of the MEBRAINS template (Balan et al. 2024), ITK-SNAP was used to manually delineate the borders of each area. Boundaries were defined based on established macroanatomical landmarks (e.g. the genu of corpus callosum to define border between ACC and aMCC) and with reference to previous identified frontal areas (e.g. premotor areas F6 and F3 to distinguish aMCC and pMCC regions; Rapan et al. 2023) to ensure anatomical consistency and accuracy. Each delineated region was then assigned its corresponding absolute receptor density values, generating volumetric receptor maps for each of the 14 receptor types within the MEBRAINS space. In addition, receptor densities were projected onto the cortical surface to create surface-based maps, enabling visualization of spatial distribution patterns across the cingulate cortex. Integration, projection, and rendering of both volumetric and surface-based receptor maps were performed using a custom Python script (v3.13).

### Statistical analysis of receptor densities

Prior to analysis, receptor density values were z-score normalized within each receptor type to account for differences in absolute density across receptor systems. Statistical analyses were performed in R (v3.6.3; R Core Team, 2020) using linear mixed-effects models (LMMs) with brain as a random effect. All p-values were corrected for multiple comparisons using the Benjamini–Hochberg false discovery rate (FDR) procedure, with significance set at FDR-adjusted p < 0.05.

For statistical (LMM-based) analyses, z-scores were computed from individual measurements for each cortical area and each brain, preserving inter-individual variability while enabling comparison across receptors. Whereas for multivariate analyses (hierarchical cluster analysis, HCA, and principal component analysis, PCA), z-scores were calculated from mean receptor densities of each area averaged across brains to ensure equal contribution of receptor types and to avoid bias from differences in absolute density values. This distinction reflects the different analytical goals of statistical and descriptive multivariate analyses.

#### Comparison of cortex on the dorsal wall of cgs and surrounding areas

To compare receptor densities between cortical areas located on the dorsal wall of the cingulate sulcus and their adjacent areas, we performed a series of linear mixed-effects model (LMM) analyses. Specifically, we compared dorsal bank areas 24cd, 24c’d, a24dd, and p24dd with (i) ventral bank cingulate areas 24cv, 24c’v, 24dv, and 23c, and (ii) adjacent dorsal cortical areas classified as prefrontal (10md, 9 m, 8Bm; Rapan et al. 2023), (pre)motor (F6, F3, 4 m; Rapan et al. 2021), and parietal cortex (TSA, PEci; Niu et al. 2025).

An initial omnibus LMM was fitted with z-scored receptor density as the dependent variable. The model included fixed effects for cortical area, receptor type, and their interaction (area × receptor), allowing assessment of overall area differences, receptor differences, and receptor-specific area effects. A random intercept for brain was included to account for repeated measurements within individual brains and to control for inter-individual variability. Significance of fixed effects was evaluated using Type-III ANOVA.

To determine whether receptor density differed across cortical areas within each receptor system, joint tests of the area effect were computed separately for each receptor type using estimated marginal means. When significant omnibus effects were detected, post-hoc pairwise comparisons of estimated marginal means were conducted to identify which specific areas differed from one another within each receptor type.

#### Revealing the intra-regional variability along the rostro-caudal axis

To assess whether receptor densities exhibited systematic variation along the rostro–caudal axis of the cingulate cortex, we analyzed z-scored receptor density values across anatomically ordered cortical areas. Each area was assigned a numerical index (ROIline) representing its location along the rostro–caudal axis of the cingulate cortex, aligned with the curvature of the corpus callosum (Giacometti et al. 2024; Loh et al. 2018).

Rostro–caudal linear trend was quantified using LMMs. Specifically, we fitted a model ROIline, receptor type, and their interaction (ROIline × receptor), allowing receptor-specific slopes to be estimated. A random intercept for brain was included to account for repeated measurements within individuals and to control for inter-individual variability.

Receptor-specific linear trends were extracted using a function, which estimates the slope of ROIline for each receptor while accounting for the mixed-effects structure of the model. These slope estimates quantify the direction (positive or negative) and magnitude of rostro–caudal gradients for each receptor type.

#### Similarities and differences in receptor fingerprints across cingulate cortex

Multivariate analyses of receptor fingerprints were performed to investigate the structural organization of the 28 identified cingulate areas. HCA was conducted to group cortical areas according to similarities in their multivariate receptor fingerprints (Palomero-Gallagher et al. 2009).

For hierarchical clustering, the normalized receptor fingerprints of each area were treated as feature vectors in a multidimensional space. Euclidean distance was used as the measure of dissimilarity between areas. Clusters were constructed using Ward’s linkage method, which iteratively merges clusters to minimize the increase in total within-cluster variance. This approach produces a hierarchical dendrogram representing nested similarity relationships among areas. Among the tested combinations of linkage criteria and distance metrics, Ward linkage with Euclidean distance yielded the highest cophenetic correlation coefficient (0.7526), indicating the best correspondence between pairwise distances in the original feature space and those implied by the dendrogram structure.

In addition to HCA, k-means clustering was performed on the same normalized receptor feature space, treating each cortical area as a single observation. The algorithm partitions areas into k non-overlapping clusters by minimizing within-cluster sum of squares (WCSS) based on Euclidean distance, assigning each area to the nearest cluster centroid. To determine the optimal number of clusters, WCSS was computed for k = 1 to 10 and plotted as a function of k. The point of maximal curvature (“elbow”) was identified using the KneeLocator algorithm (Satopaa et al. 2011) implemented in an in-house Python (v3.13) script, providing an objective estimate of the optimal cluster number.

Finally, principal component analysis (PCA) was applied to the same normalized receptor feature space to reduce dimensionality and facilitate visualization. Areas were projected onto the first two principal components, allowing visualization of their distribution in reduced-dimensional space. The proportion of total variance explained by each principal component was indicated along the corresponding axes.

#### Receptor-driven difference between anatomically defined cingulate regions

In addition to the dorsal bank analyses (2.5.1), we applied the same statistical framework to examine receptor density differences across individual cingulate areas. Using LMMs, we tested whether receptor density varied as a function of cortical area, receptor type, and their interaction, while accounting for repeated measurements within brains through inclusion of a random intercept for each macaque brain. To further investigate an anatomical organization, receptor densities from all cingulate areas were subsequently grouped into four predefined cortical regions (ACC, aMCC, pMCC, and PCC) based strictly on anatomical boundaries, independent of clustering results. Differences in receptor profiles were then assessed at the regional level.

An omnibus linear mixed-effects model was fitted with z-scored receptor density as the dependent variable. The model included fixed effects for region, receptor type, and their interaction (region × receptor), allowing assessment of overall regional differences, receptor differences, and receptor-specific regional effects. A random intercept for brain was included to account for within-subject correlation and inter-individual variability. Significance of fixed effects was evaluated using Type-III ANOVA.

To determine whether receptor density differed between anatomical regions within each receptor system, joint tests of the region effect were performed separately for each receptor type using estimated marginal means. When significant omnibus effects were detected, post-hoc pairwise comparisons were conducted to identify which specific regions differed from one another within each receptor.

## Results

**Fig. 2:**
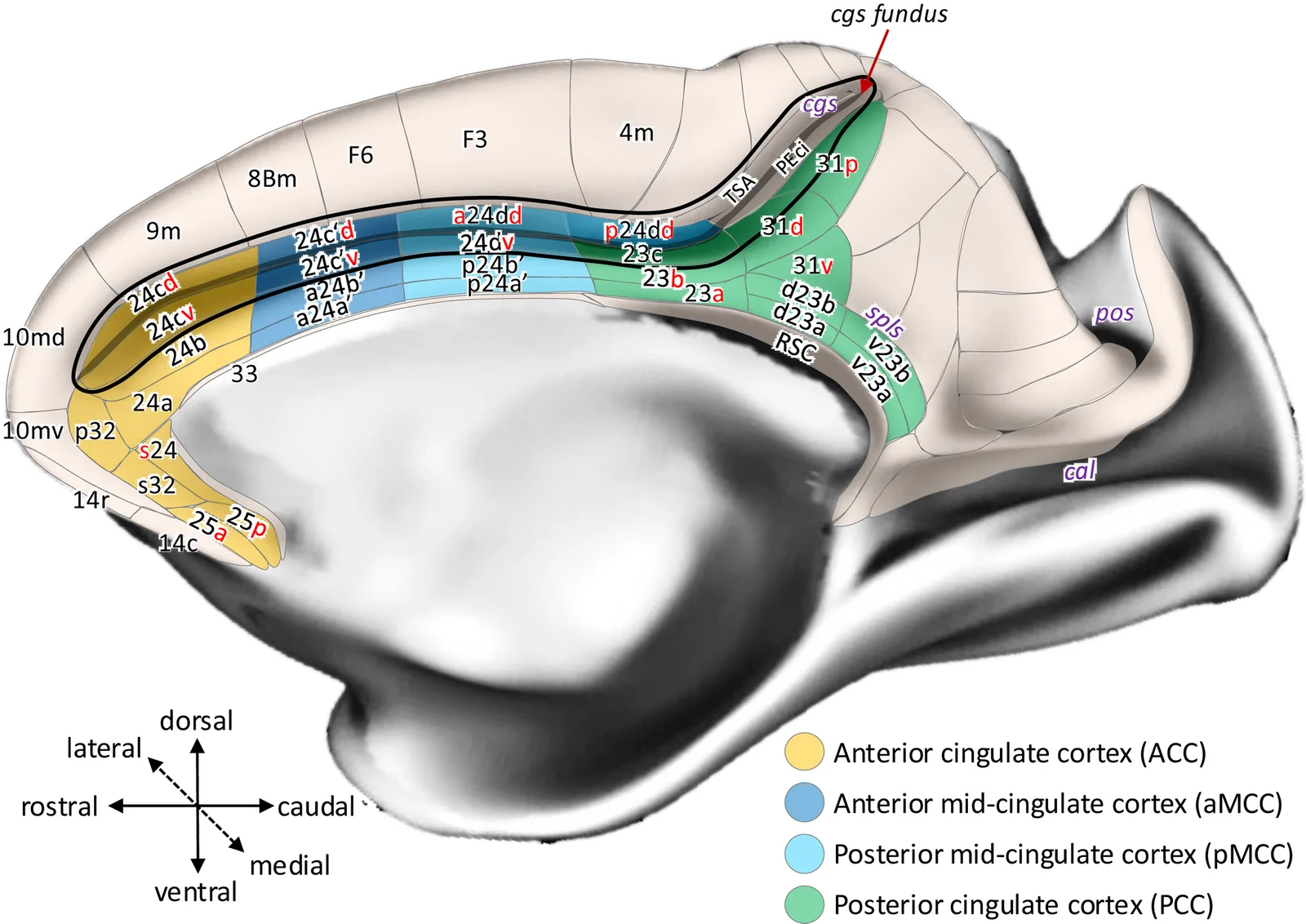
Map of the medial surface of a macaque hemisphere showing the location and extent of cyto- and receptor architectonic areas of the cingulate cortex. The 28 identified cingulate areas are color-coded by region: ACC (orange), aMCC (dark blue), pMCC (light blue), and PCC (green). Surrounding prefrontal, (pre)motor, parietal, retrosplenial and occipital areas are shown in pale yellow. Black lines indicate edges of the cgs, and transparent black line mark fundus of the sulcus (additionally marked by the red arrow). *cal* calcarine sulcus, *cgs* cingulate sulcus, *spls* splenial sulcus, *pos* parieto-occipital sulcus

We identified 28 cyto- and receptor architectonically distinct areas that could be assigned to the ACC, aMCC, pMCC and PCC regions, and generated comprehensive surface-based (Figs. 2 and 9) and volume-based (Fig. S5) maps of the macaque cingulate cortex.

### Cytoarchitecture of cingulate cortex

#### ACC

Within the ACC, we confirmed the existence of areas 24b, 24c (here referred to as 24cv), p32 and s32, and found subdivisions in Vogt’s (Vogt et al. 2005) areas 25 (25a and 25p) and 24a (24a and s24). In addition, we identified cingulate area 24cd on the dorsal wall of the cgs.

**Fig. 3:**
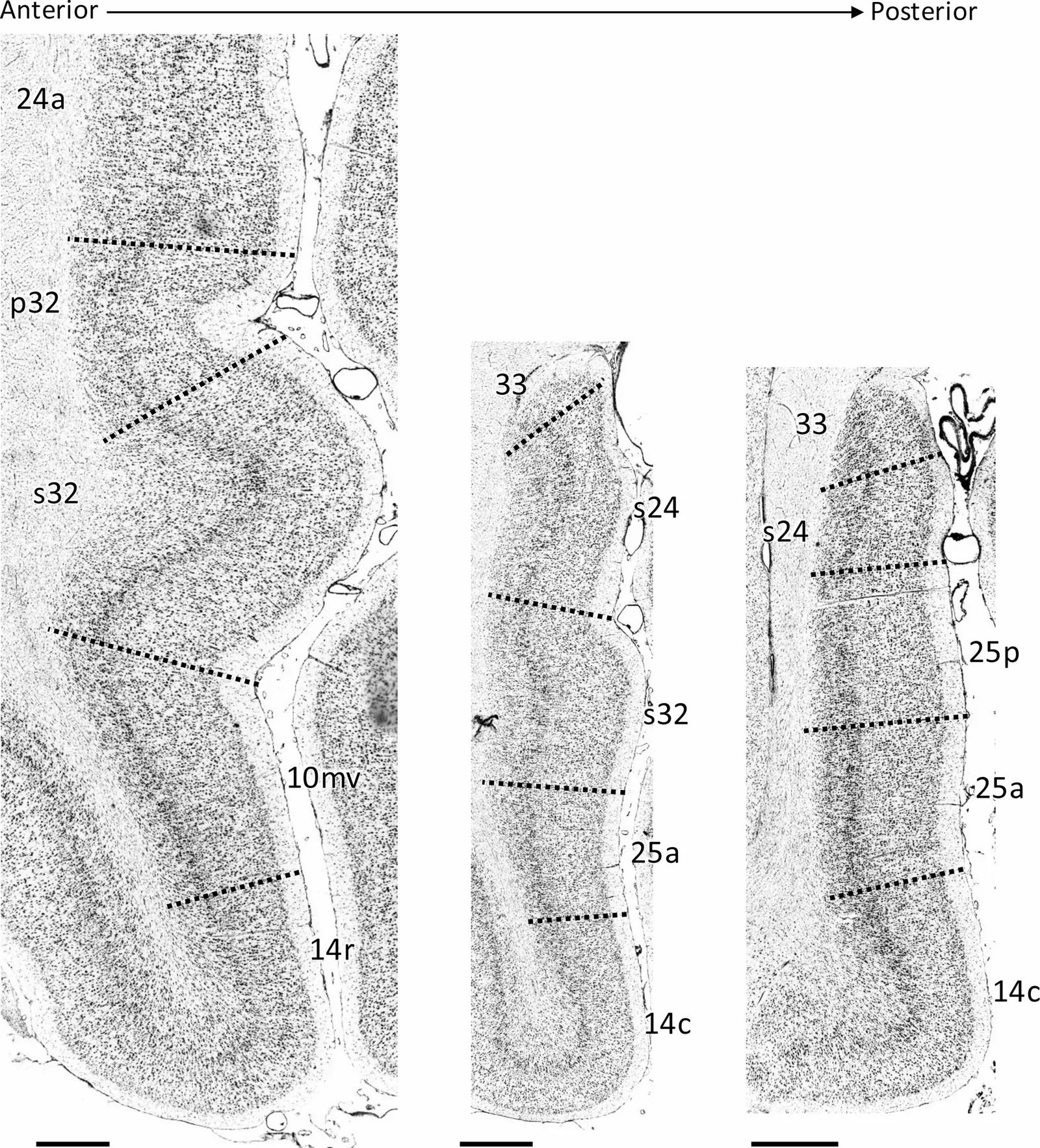
Cytoarchitecture of ACC areas 32, 25 and s24. High-resolution images of coronal sections through the pregenual (left) and subgenual (middle and right) regions of the cingulate cortex, located rostral to and immediately below the genu of the corpus callosum, respectively. It shows the cytoarchitecture of ACC areas 25a, 25p, p32, s32 and s24 and of adjacent orbitofrontal areas 14r and 14c. Black dotted lines indicate borders between neighboring areas. Scale bar is 1cm. *cgs* cingulate sulcus

Area p32 occupies posterior portion of the frontal pole, whereas s32 is located within the subcallosal cortex. Unlike the rest of ACC areas, p32 and s32 exhibit a thin layer IV and therefore represent a typical dysgranular cortex (Fig. 3). This feature also distinguishes them from the adjacent granular prefrontal areas 10md and 10mv.

Areas 25a and 25p, also located within the subcallosal region, are periallocortical in nature and thus clearly distinguishable from surrounding neocortical areas 32, 10mv and 14c, mainly due to the fact they present a layer IV, and proisocortical area 24 mainly due to its differentiated layer III. They can also be clearly delineated from allocortical area 33 due to its even more primitive laminar appearance (Fig. 3). Areas 25a and 25p both have a poor laminar organization, with a wide cortical lamina formed by supragranular layers (II-III) and a narrower, but denser, lamina formed by infragranular layers (V-VI). Cytoarchitectonic differences between 25a and 25p (Fig. 3):

- In area 25p, layer II is thin, but clearly distinguishable from layer III, while in area 25a these layers are not separable and are therefore labeled as layer II–III.
- In area 25a, but not in 25p, layer V can be clearly subdivided into sublayers Va and Vb due to the lower packing density in the latter. Consequently, the border between layers V and VI is more distinct in 25a than in 25p.

Area s24 is located below the genu of the corpus callosum, whereas 24a is found rostral and dorsal to this macroanatomical landmark, and is delimited dorsally by area 24b. Areas s24 and 24a can be distinguished based on differences in layers II and III:

- Layer II is wider and more densely populated in 24a than in s24.
- The border between sublayers IIIa and IIIb was more clear in s24.

**Fig. 4:**
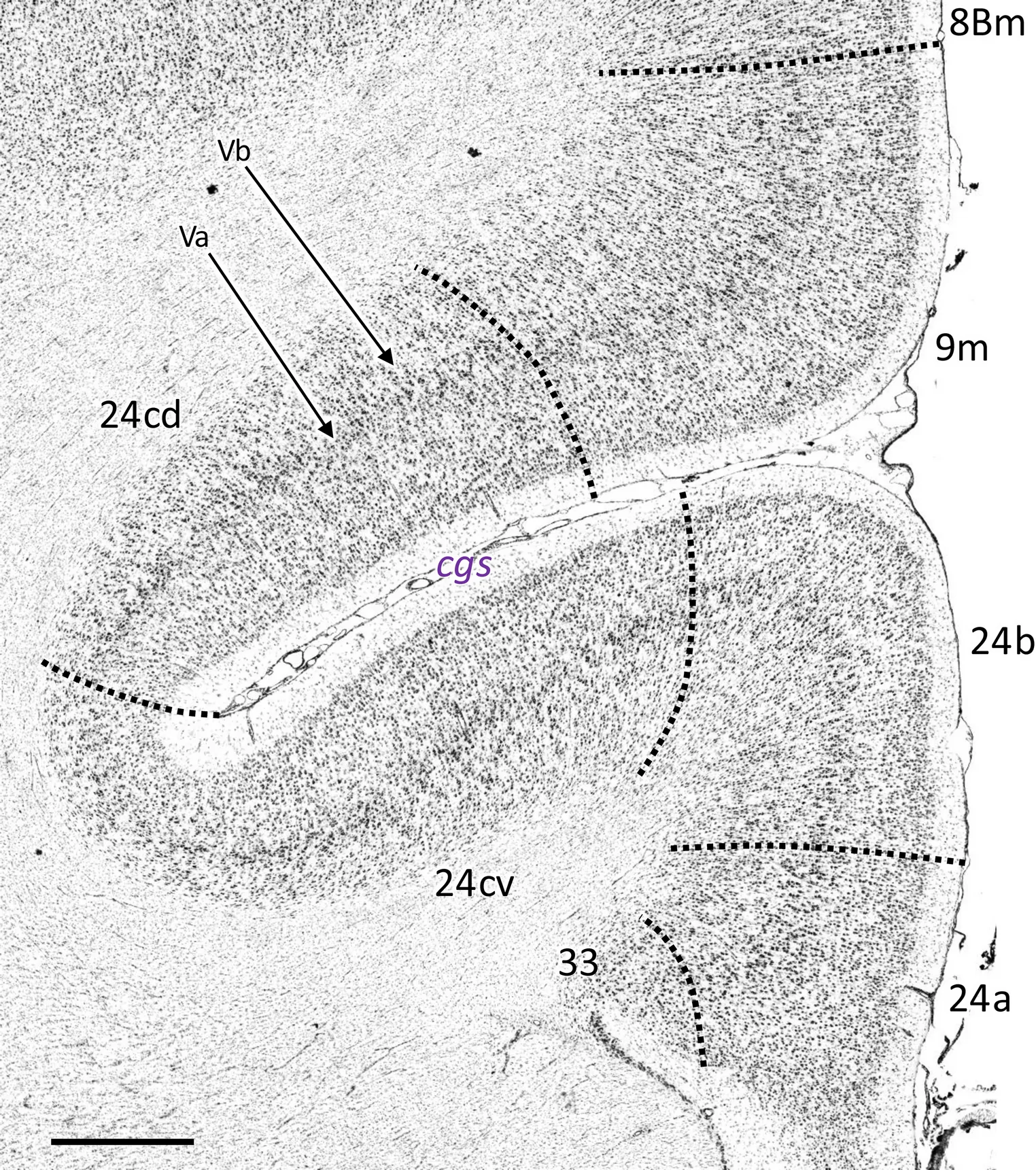
Cytoarchitecture of ACC areas 24. High-resolution image of a coronal section through the cingulate gyrus and sulcus just caudal to the genu of the corpus callosum. It shows the cytoarchitecture of ACC areas 33, 24a, 24b, 24cv, 24cd and of adjacent prefrontal areas 9 m and 8Bm. Black dotted lines indicate borders between neighboring areas. Black dotted lines indicate borders between neighboring areas. Scale bar is 1cm. cgs—cingulate sulcus**.** The statistically defined borders of the newly identified ACC area 24cd and its adjacent areas 24cv and 9 m are shown in Fig. S1

We identified an area 24c on the cingulate bank of the cingulate sulcus, but here adopted the term 24cv, because we found the cortex on the dorsal bank to differ from 24cv, but to also present a cingulate-like cytoarchitecture. Specifically, area 24cd was characterized by (Fig. 4):

- Area 24cd is agranular, whereas prefrontal areas all present a clear layer IV.
- Layer VI in area 24cd is more densely populated than in area 9 m or in area 24cv, resulting in a sharper border between layer VI and the underlying white matter
- Larger pyramids in layer Va of area 24cd appear to be more scattered than the ones in 24cv.
- Layer Vb is paler in area 24cd than in area 24cv, where it is populated by medium-size pyramids.

#### MCC

Within the MCC, we confirmed Vogt et al. (2005) anterior (aMCC) areas a24a′ and a24b′ on the gyral surface, as well as a24c′ (here designated 24c′v) on the ventral bank of the cingulate sulcus. In addition, we identified area 24c’d on the dorsal wall of the cgs. Caudal to aMCC we identified his posterior (pMCC) areas p24a′ and p24b′ on the gyrus and area 24 d (here designated 24dv) within the ventral bank of the cgs. Again, we found the dorsal bank of the cgs to contain cingulate cortex, namely area a24dd rostrally and area p24dd caudally (Fig. 2).

**Fig. 5:**
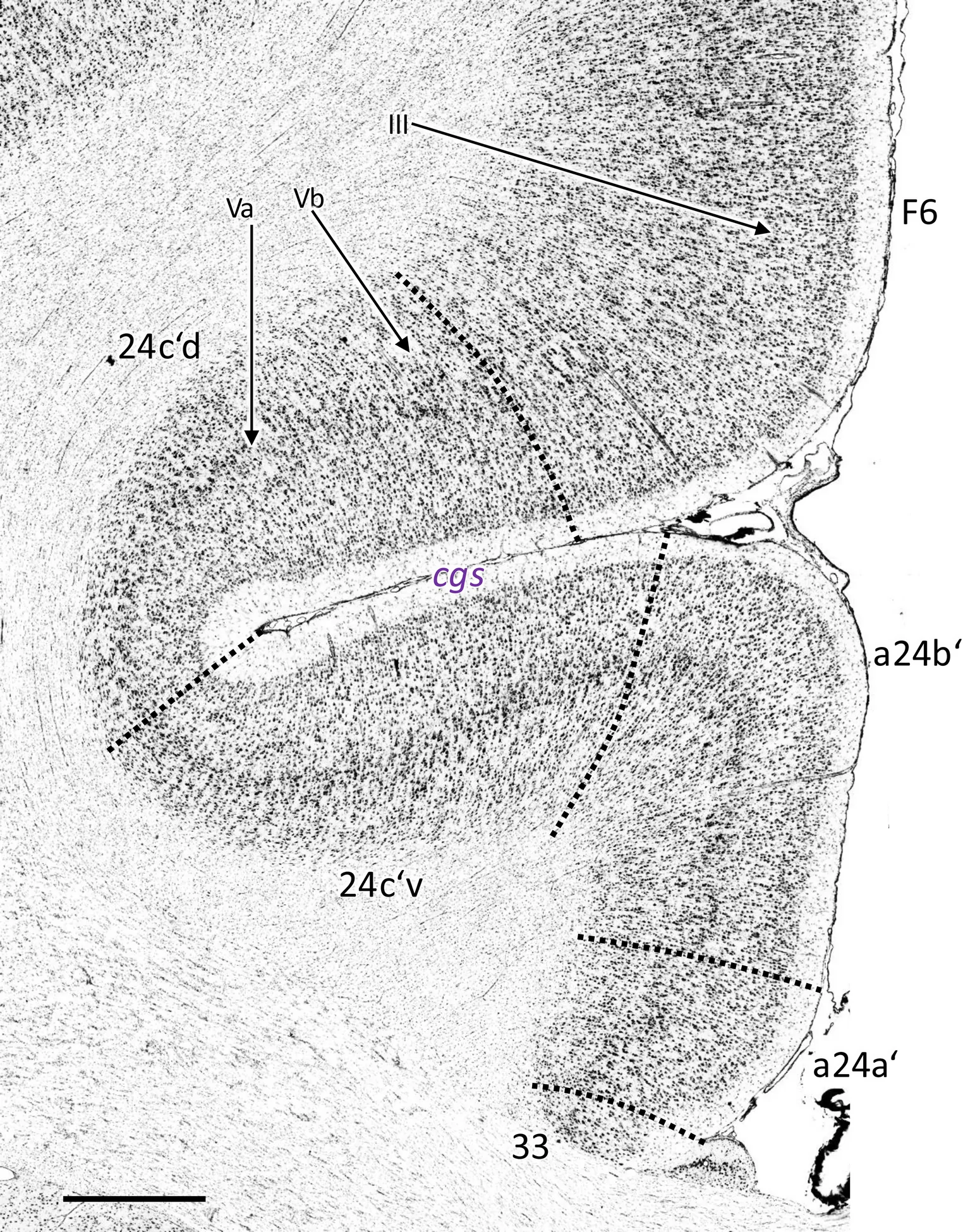
Cytoarchitecture of aMCC areas. High-resolution image of a coronal section through the cingulate gyrus and sulcus caudal to the ACC. It shows the cytoarchitecture of aMCC areas 33, a24a’, a24b’, 24c’v, 24c’d and of adjacent premotor area F6. Black doted lines indicate borders between neighboring areas. Roman numerals denote cortical layers. Scale bar is 1cm. cgs—cingulate sulcus. The statistically defined borders of the newly identified aMCC area 24c’d and its adjacent areas 24c’v and F6 are shown in Fig. S2

**Fig. 6:**
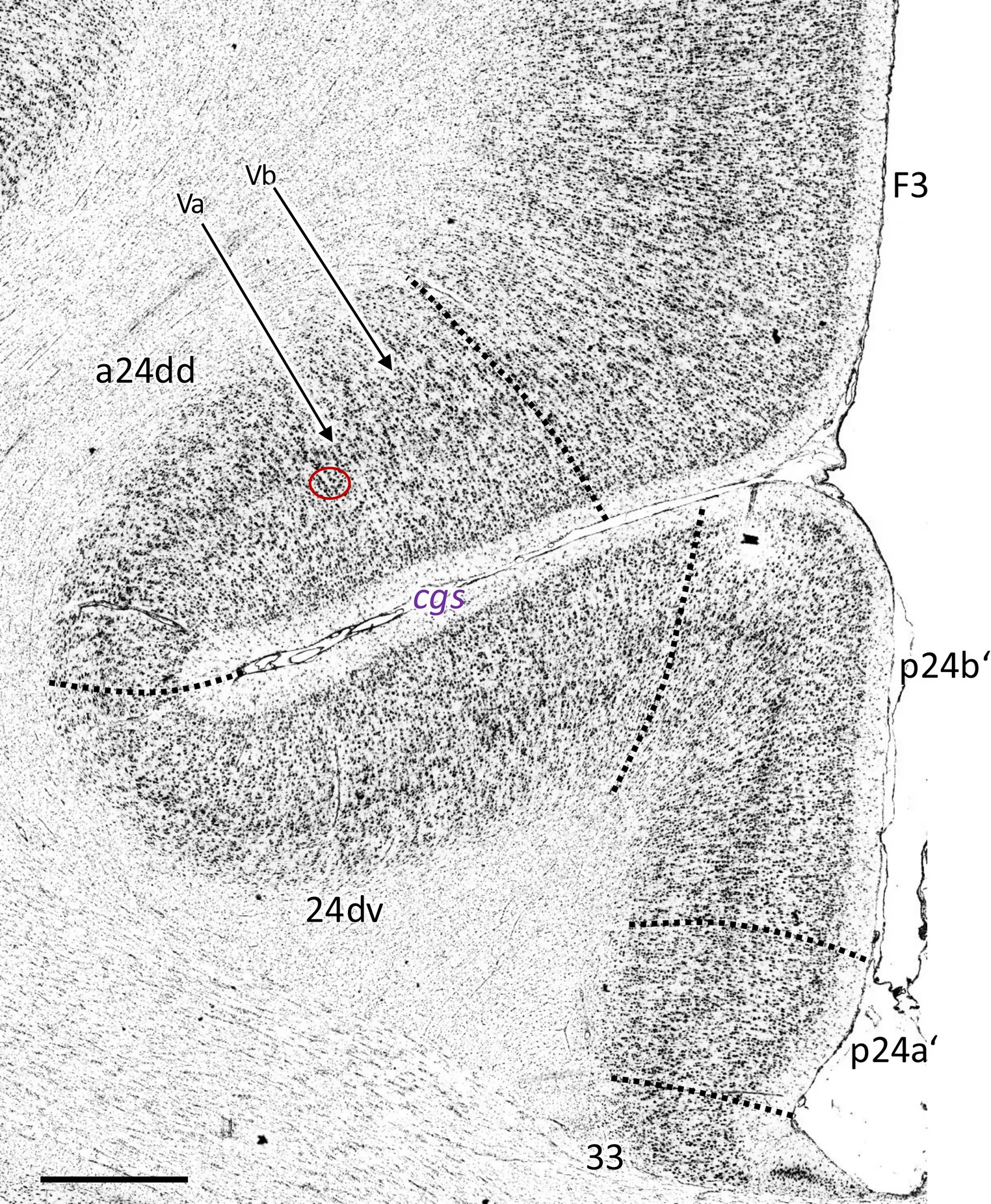
Cytoarchitecture of pMCC areas. High-resolution image of a coronal section through the cingulate gyrus and sulcus caudal to the aMCC. It shows the cytoarchitecture of aMCC areas 33, p24a’, p24b’, 24dv, a24dd and of adjacent premotor area F3. Black doted lines indicate borders between neighboring areas. Red circles highlight characteristic clusters of pyramidal neurons in a deep layer III within area a24dd. Roman numerals denote cortical layers. Scale bar is 1cm. cgs—cingulate sulcus**.** The statistically defined borders of the newly identified pMCC area a24dd and its adjacent areas 24dv and F3 are shown in Fig. S3

Similar to the rostrally adjacent ACC subdivisions of area 24, all MCC areas belong to the agranular cortex, and likewise exhibit dorso–ventral differences in cytoarchitecture, particularly in layers II and Va, and reflected in the suffixes a, b and c. In general terms, layers II, III and V of aMCC areas are populated by larger neurons than the respective areas in ACC. Cytoarchitectonic differences between aMCC and pMCC are mainly found in layers III and Va (Figs. 5 and 6):

- Deep layer III presents occasional cell clusters in areas of pMCC but not in aMCC.
- Layer Va of pMCC areas contains slightly smaller and more densely packed pyramids than that of corresponding aMCC areas. Thus, the sublamination of layer V is not as prominent in pMCC as in ACC, aMCC or PCC.

The gyral cortex of the midcingulate region is considerably narrower than that of the adjacent premotor and motor cortex. The thickness of the cortex on the dorsal bank of the cgs increases slightly when moving from the fundus to the surface of the hemisphere. Thus, although areas 24c’d, a24dd and p24dd have a conspicuous layer Va which clearly differentiates them from the adjacent (pre)motor cortex, this layer appears broadened and partially distorted when compared with other cingulate areas, likely reflecting local sulcal geometry. Both a24dd and p24dd share a distinctive cytoarchitectonic feature, namely the presence of occasional clusters of pyramidal neurons in deep layer III (circled in Figs. 5 and 6), a characteristic not observed in surrounding cortical areas. In addition, p24dd exhibits a more densely packed layer VI and a sharper border with the white matter than adjacent motor areas.

#### PCC

**Fig. 7:**
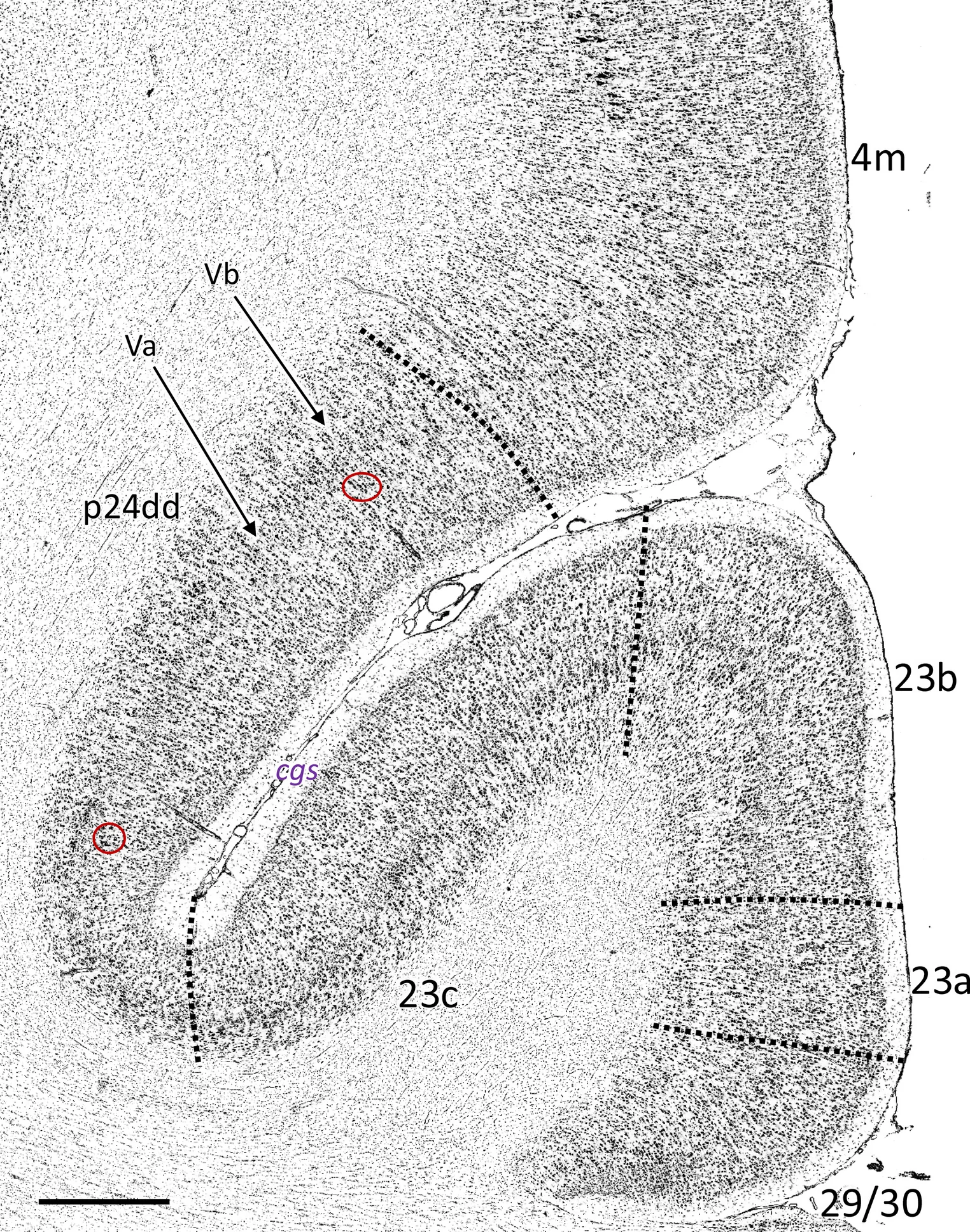
Cytoarchitecture of dysgranular PCC areas and pMCC area p24dd. High-resolution image of a coronal section through the cingulate gyrus and sulcus just below primary motor cortex. It shows the cytoarchitecture of PCC areas 23a, 23b, 23c, of pMCC area p24dd, and of adjacent retrospelenal areas 29 and 30, and primary motor area 4m. Black dotted lines indicate borders between neighboring areas. Red circles highlight characteristic clusters of pyramidal neurons in a deep layer III within area p24dd, and black arrows indicate its layers Va and Vb. Roman numerals denote cortical layers. Scale bar in is 1cm. *cgs* cingulate sulcus**.** The statistically defined borders between the newly identified pMCC area p24dd and its adjacent areas 23c and 4 m are shown in Fig. S4

**Fig. 8:**
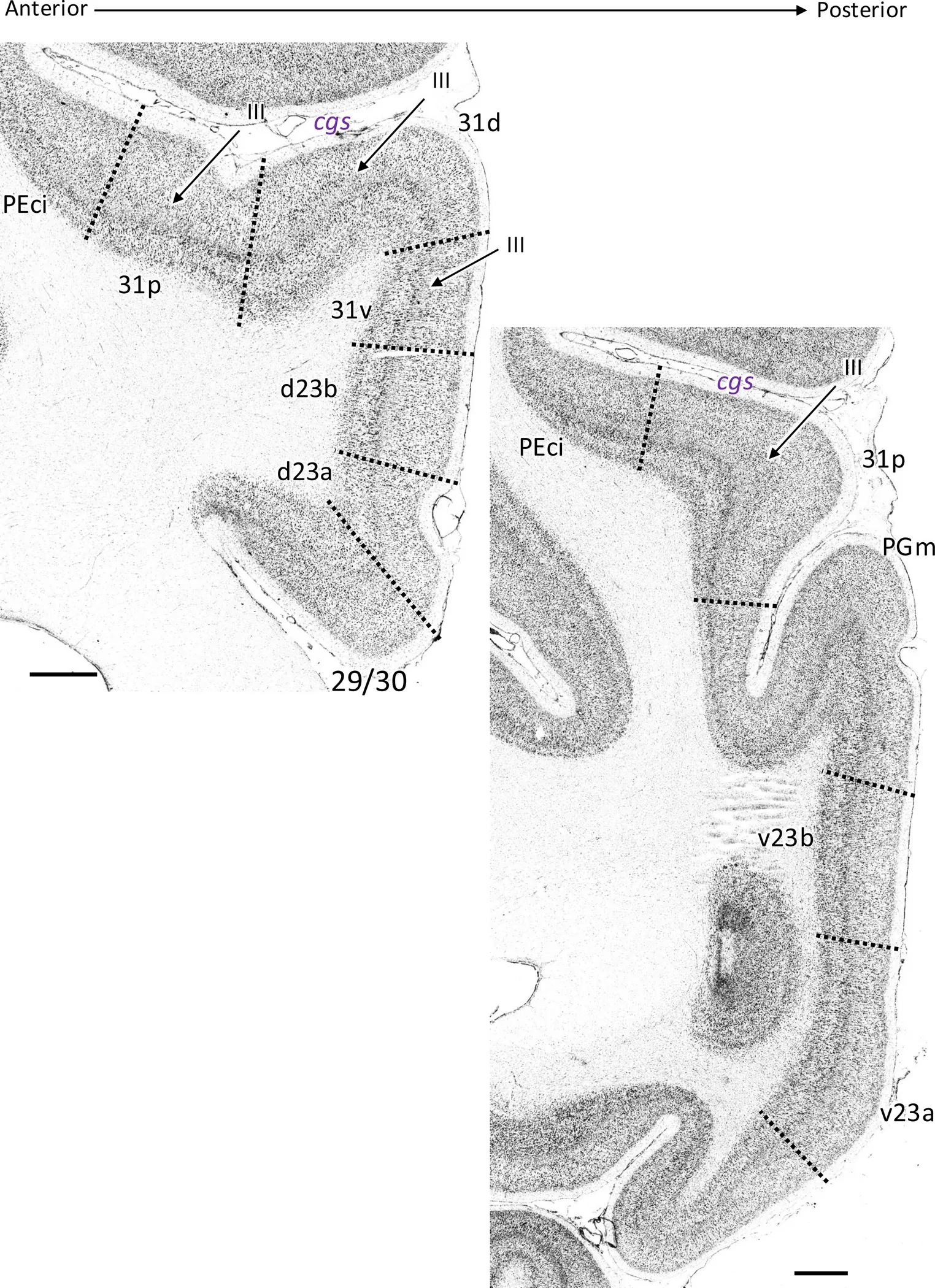
Cytoarchitecture of granular PCC areas d23, v23 and 31. High-resolution images of coronal sections through the cingulate gyrus and sulcus caudal to the primary motor cortex (left) and more posteriorly at the border with the parietal cortex (right). It shows the cytoarchitecture of PCC areas d23a, d23b, v23a, v23b, 31 d, 31v and 31p, and of adjacent retrospelenal areas 29/30, and parietal areas PEci and PGm. Black dotted lines indicate borders between neighboring areas. Black arrows highlight characteristic layer of pyramidal neurons in a deep layer III within areas 31. Roman numerals denote cortical layers. Scale bar in is 1cm. *cgs* cingulate sulcus

Within the PCC we confirmed Vogt’s (Vogt et al. 2005) areas d23a, d23b, v23a, v23b and 23c, and found subdivisions of his area 23 d (23a and 23b) and of Morecraft et al. (2004) area 31 (31d, 31v and 31p). Areas 23a, 23b and 23c are dysgranular, whereas the remaining PCC areas display a particularly well-developed and densely populated layer IV (Figs. 7 and 8).

The main cytoarchitectonic characteristics enabling the identification of areas within the dysgranular PCC areas are as follows (Fig. 7):

- The prominence of layer IV decreases gradually when moving from 23a, through 23b and onto 23c. Thus, 23a is the most dysgranular of the three areas.
- In contrast, the prominence of layer Va increases gradually when moving from 23a, through 23b and onto 23c. Thus, 23c presents the most densely packed layer Va of the three areas.
- Areas 23b and 23c, but not 23a, display a gradual increase in cell packing density with increasing depth of layer III.
- Layer Va is the least prominent in 23a, where almost no large pyramids are found, compared to 23b and 23c

We identified three subdivisions within area 31; 31 d is located within the cgs posterior to 23c and extends onto the gyral surface. It is delimited caudally by area 31p, which reaches the caudal-most portion of the cgs. Area 31v is found ventral to 31 d and dorsal to d23b. All three areas exhibited a sharply defined boundary between layers II and III, resulting from reduced neuronal density in the upper portion of layer III, and which distinguished them from neighboring areas (23c, d23b, PEci, and PGm). Moreover, across area 31, pyramidal neurons in deep layer III were similar in size to, or larger than those in layer Va, distinguishing these areas from other cingulate areas. The main cytoarchitectonic differences between subdivisions of area 31 are (Fig. 8):

- Pyramidal neurons in layer Vb are evenly distributed in 31 d but appeared in clusters in 31v.
- Cell packing density is higher in areas 31 d and 31v than in 31p, and this is particularly true for layer Va.

### Receptor architecture of cingulate cortex

#### Size and shape of receptor fingerprints differ between areas and across regions

**Fig. 9:**
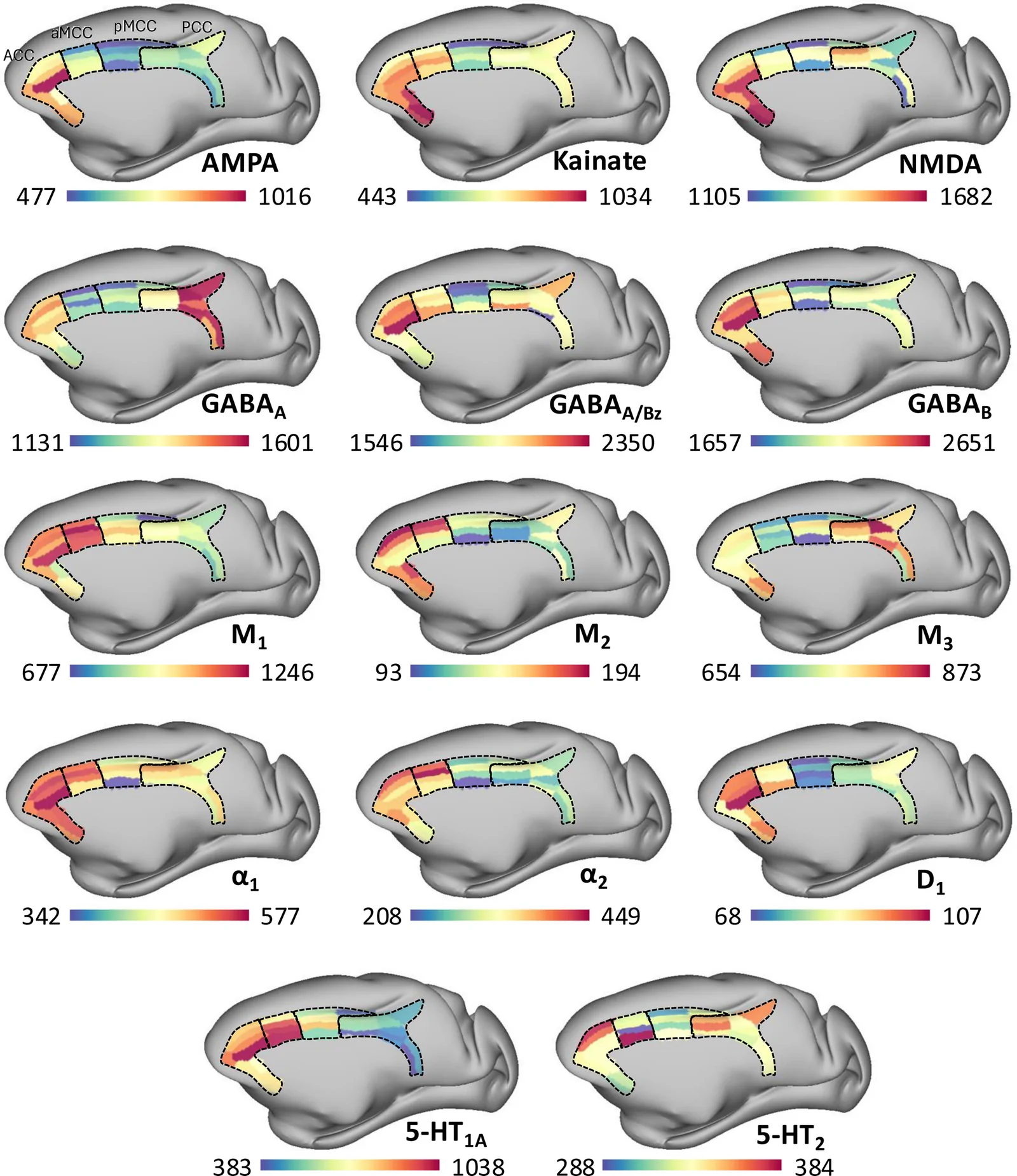
Surface representation of a macaque hemisphere showing the location and extent of cyto- and receptor architectonic areas of the cingulate cortex. The 28 identified areas are color-coded according to their absolute mean densities across the 14 receptor types analyzed. Dashed lines indicate borders of the molecular regions (ACC, aMCC, pMCC and PCC; as shown in the surface distribution of the AMPA receptor). Scale bar represents absolute mean values of receptor densities (fmol/mg protein). The volumetric representation of this figure is shown in Fig. S5

**Fig. 10:**
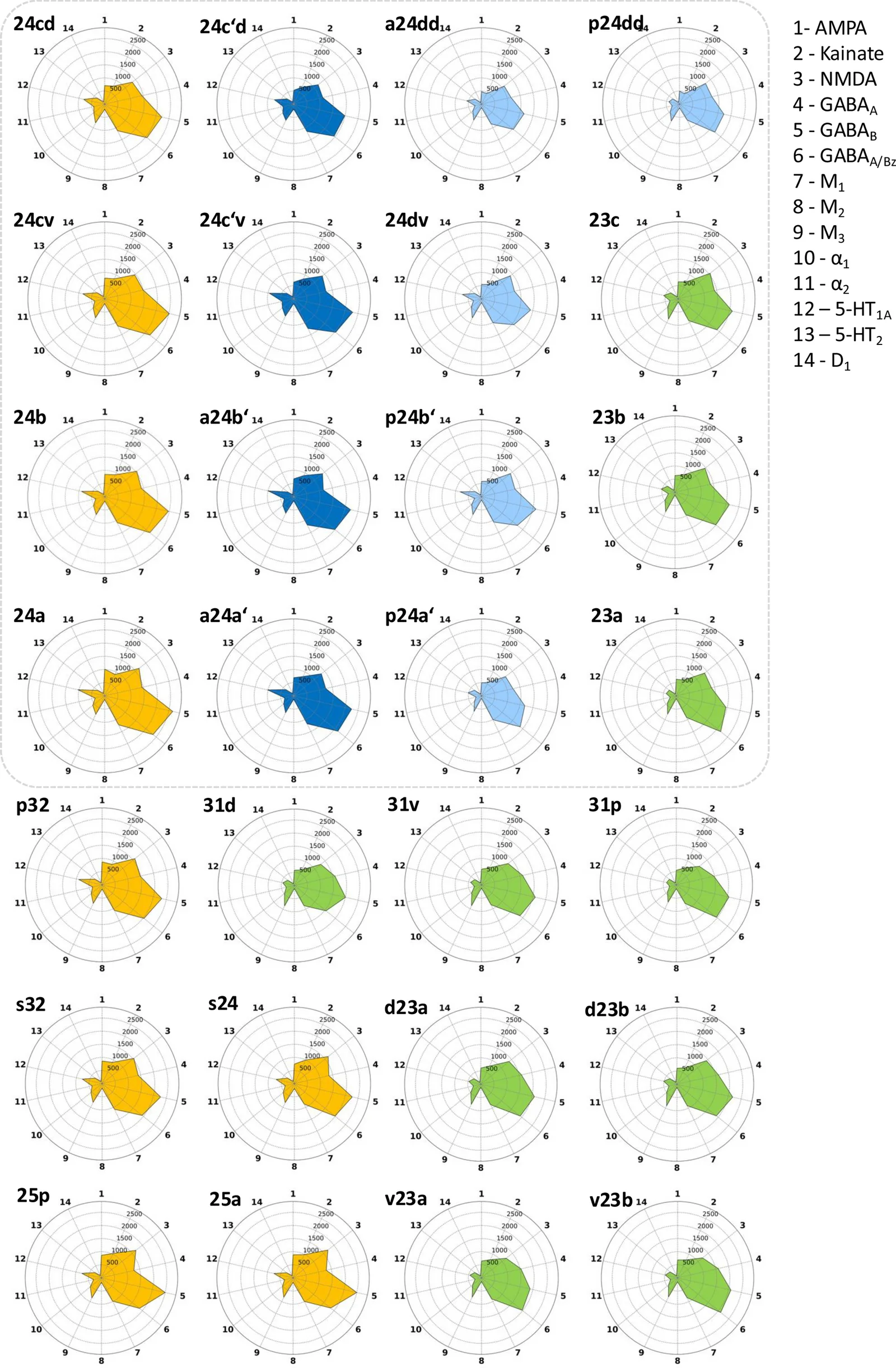
Absolute receptor fingerprints of the 28 identified cingulate areas. Each radial plot shows the absolute mean densities (in fmol/mg protein) of the respective receptor types for each area. Receptor types are numbered around the plots and correspond to the legend in the top right. Axes are scaled identically across all area plots. Areas within the dashed square represent fingerprints of subdivisions of areas 24 at the level of ACC (orange), aMCC (dark blue), pMCC (light blue), and dysgranular areas 23 at the level of PCC (green), highlighting rostro–caudal differences along the cingulate gyrus and within the cingulate sulcus (see Figs. 4, 5, 6 and 7). The remaining ACC and PCC areas are shown below the square in respective colors

Defined areas can be differentiated by distinct receptor density patterns across the cortex, which we displayed on the surface-based (Fig. 9) and volume-based (Fig. S5) versions of the MEBRAINS template. ACC areas generally displayed the highest receptor densities across the analyzed receptors, whereas overall lowest densities were found in pMCC areas, which was reflected in their generally largest and smallest receptor fingerprints, respectively (Table 1; Figs. 9 and 10, Fig. S8). Within ACC area 24a most frequently exhibited the highest receptor densities, and within pMCC areas a24a’ and a24dd the lowest densities. Notably, M_1_, M_2_, α_2_, and 5-HT_1A_ receptors reached their peak concentrations in aMCC, and highest overall GABA_A_ and M_3_ densities were found in PCC. The densities of kainate, NMDA and M_3_ receptors were higher in the subcallosal areas of ACC than in those rostral or dorsal to the corpus callosum, and the opposite was true for GABA_A_, M_1_ and α_2_ receptors. MCC than in the pMCC areas. Interestingly, for most receptors, densities were higher in the aMCC than in the pMCC areas. Interestingly, the smallest degree of differentiation concerning receptor densities was found within the PCC with the exception of the GABA_A_ and M_3_ receptors mentioned above.

**Table 1:** Absolute receptor densities (mean over all layers ± s.d.) in fmol/mg protein. s.d. – standard deviation

Area	AMPA	Kain	NMDA	GABA_A_	GABA_B_	GABA_A/Bz_	M_1_	M_2_	M_3_	α_1_	α_2_	5HT_1A_	5HT_2_	D_1_
25p	861	1034	1682	1272	2498	1860	982	172	848	546	307	769	320	99
s.d	132	92	229	299	386	273	327	54	158	47	77	204	57	22
25a	879	984	1701	1302	2497	1840	995	167	820	519	333	751	311	94
s.d	146	85	223	308	250	320	317	58	120	63	68	204	75	31
s24	743	1000	1655	1354	2272	1984	864	186	805	547	356	716	336	95
s.d	159	61	216	301	297	95	272	45	49	42	61	205	58	28
s32	860	898	1543	1405	2278	1942	1084	170	793	541	390	769	331	89
s.d	160	84	217	177	257	506	328	36	112	83	114	246	88	21
p32	868	860	1590	1484	2321	2035	1072	155	773	528	369	910	342	89
s.d	160	84	217	177	257	506	328	36	112	83	114	246	88	21
24a	1016	901	1670	1451	2651	2350	1218	134	776	577	371	1038	334	107
s.d	64	43	161	208	293	354	225	28	43	32	51	291	77	26
24b	845	921	1547	1418	2469	2178	1089	156	752	545	352	902	333	101
s.d	154	72	309	234	358	312	315	34	99	100	68	271	60	28
24cv	785	840	1457	1498	2517	2200	1143	178	802	557	384	850	367	99
s.d	125	66	275	171	503	527	246	38	65	76	96	137	85	31
24cd	706	760	1343	1451	2222	2056	1126	194	798	527	421	816	379	99
s.d	135	72	290	195	283	417	224	39	70	78	55	111	68	34
a24a'	690	815	1343	1248	2255	2148	1174	129	710	486	350	1015	384	97
s.d	172	78	184	396	294	172	273	43	127	66	53	273	73	31
a24b'	678	891	1399	1152	2216	1999	1190	154	695	534	368	1002	288	87
s.d	179	103	312	349	190	420	254	49	165	92	100	296	35	44
24c'v	633	834	1390	1255	2295	2036	1246	183	733	554	449	954	328	89
s.d	190	90	368	368	190	313	259	40	165	71	86	233	46	40
24c'd	529	718	1186	1131	1991	1961	1158	189	689	533	411	754	323	91
s.d	164	91	316	305	295	374	222	43	181	57	66	172	30	32
p24a'	494	563	1168	1226	1686	1881	1001	93	654	342	208	525	336	71
s.d	251	178	295	203	534	531	250	39	135	121	62	162	42	13
p24b'	578	649	1406	1301	2121	1742	1066	115	752	478	265	829	310	69
s.d	208	139	215	204	459	532	281	34	125	101	75	223	52	11
24dv	545	604	1399	1283	1910	1575	1008	135	806	492	311	747	325	74
s.d	202	80	118	178	533	426	255	37	69	61	78	101	43	14
a24dd	477	449	1105	1148	1657	1547	837	130	680	423	253	574	301	68
s.d	184	71	192	205	628	431	230	34	73	65	67	121	39	19
p24dd	493	482	1263	1329	1785	1717	735	141	716	449	284	408	323	76
s.d	91	78	301	311	336	438	227	45	100	50	30	82	76	9
23a	627	662	1387	1447	1946	2172	910	102	764	433	237	395	344	82
s.d	163	53	270	308	374	637	274	33	155	44	35	108	74	15
23b	636	734	1466	1447	2221	1994	951	103	825	487	257	542	367	79
s.d	174	66	268	233	414	624	293	34	167	28	45	159	88	14
23c	644	708	1550	1466	2174	1884	919	112	852	512	321	564	356	80
s.d	149	74	208	258	414	533	321	32	142	41	33	117	79	15
d23a	612	763	1382	1593	2102	1927	858	121	840	490	288	471	333	80
s.d	136	125	114	180	373	483	320	44	126	37	40	135	85	16
d23b	603	689	1432	1612	2170	1916	898	121	906	495	311	518	338	88
s.d	98	84	125	178	238	406	331	39	115	25	29	141	87	15
v23a	640	768	1198	1538	1890	1978	885	140	817	433	249	450	327	85
s.d	131	135	258	407	335	208	339	56	150	47	37	128	80	25
v23b	682	760	1222	1593	2090	2109	833	149	808	440	273	481	362	86
s.d	12	83	249	367	306	185	291	40	123	35	26	131	76	26
31d	577	664	1294	1592	2007	1546	852	116	864	479	314	451	321	76
s.d	98	116	146	170	371	297	256	45	163	36	33	80	80	15
31v	593	718	1295	1601	2094	1871	834	121	873	472	273	421	322	80
s.d	82	119	94	176	299	431	299	30	129	25	23	120	79	17
31p	550	707	1125	1473	2068	1966	780	143	729	430	259	383	335	82
s.d	92	80	365	400	336	267	252	44	111	31	55	41	71	23

Receptor distributions revealed not only rostro–caudal differences along the cingulate cortex, but also a dorso–ventral segregation when moving away from the corpus callosum (Fig. 9 and Fig. S5). These differences were more pronounced in the ACC and MCC regions than in the PCC. For M_2_ and GABA_A/Bz_, this segregation followed a consistently graded pattern. Specifically, M_2_ receptor densities were lowest in subdivisions of area 24 located immediately dorsal to area 33 and increased stepwise toward subdivisions of area 24 along the cingulate sulcus (cgs). In contrast, GABA_A/Bz_ receptors displayed the opposite gradient, with lower densities in cgs areas relative to more ventral ones. Finally, among all receptor types examined, 5-HT_2_ displayed the highest degree of regional variability, exhibiting a heterogeneous distribution pattern across cingulate areas.

The newly identified cytoarchitectonic subdivisions of areas 25, 24 and 31 were also confirmed by differences in their receptor architecture. Specifically, area 25a contained higher α_2_ receptor densities relative to 25p, and the opposite pattern was observed for M_2_, M_3_, and α_1_ receptors. Interestingly, the fingerprint of s24 more closely resembled that of areas 25a and 25p than that of 24a (Fig. 10, Fig. S8), and the subdivisions of area 24 within the cgs will be described in detail below. Area 31 d showed the most distinct receptor fingerprint compared to 31v and 31p, mainly due to its considerably lower GABA_A/Bz_ density (Table 1, Fig. 10 and Fig. S8).

#### Does the dorsal bank of the cingulate sulcus contain cingulate cortex?

**Fig. 11:**
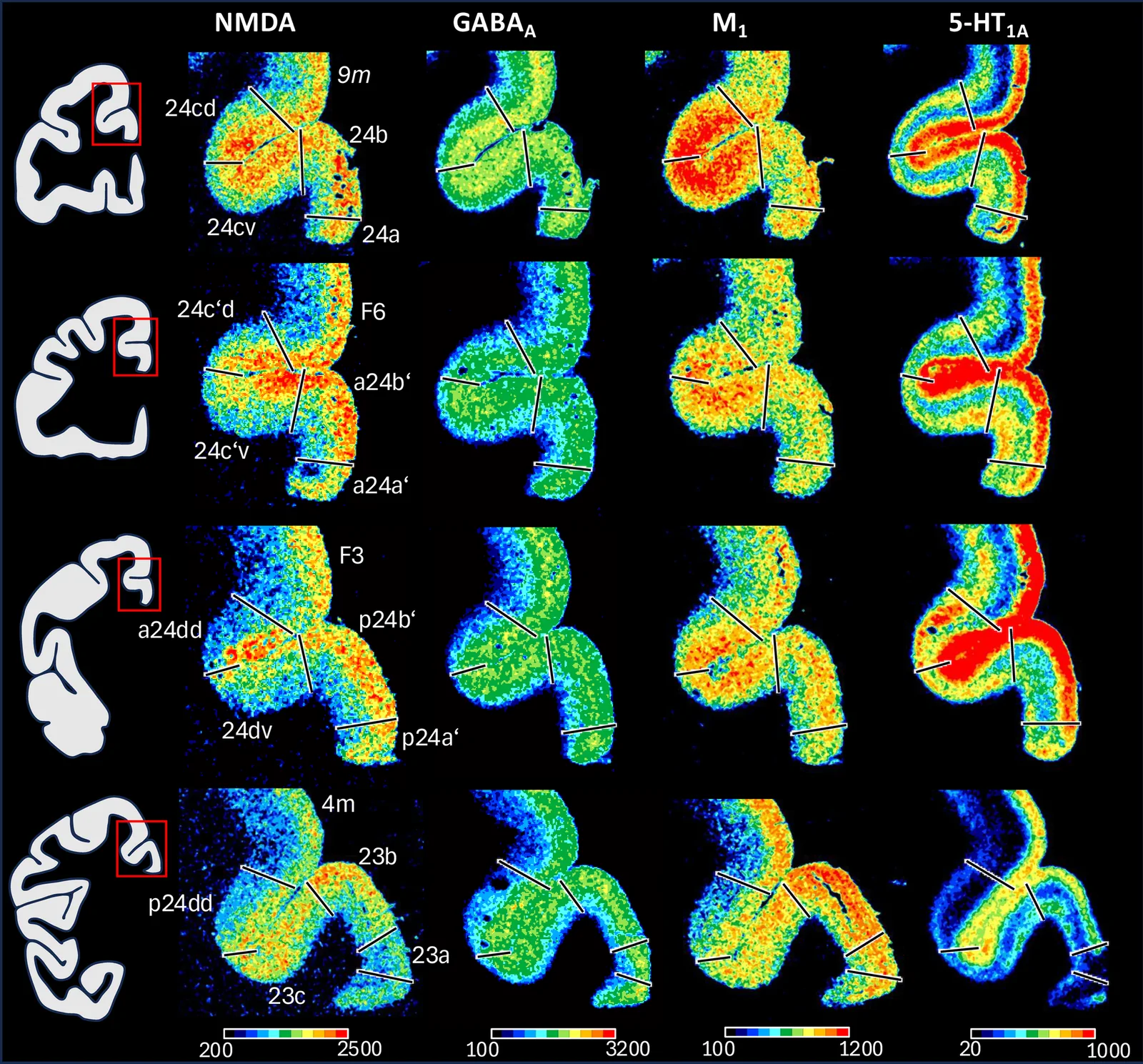
Laminar receptor distribution patterns. On the left, schematic drawings of coronal sections processed for receptor labeling illustrate the positions of cingulate region of interest (indicated by red squares) at different rostro–caudal levels: ACC (first row), aMCC (second row), pMCC (third row), and PCC (fourth row). The PCC level shows dysgranular areas 23 together with pMCC area p24dd. At each level, the respective adjacent frontal areas are also shown. On the right, corresponding exemplary section cuts depict the laminar distribution of NMDA, GABA_A_, M_1_, and 5-HT_1A_ receptors within the regions of interest. Lines indicate borders between the defined cingulate areas. Scale bars represent receptor densities (fmol/mg protein)

To answer this question, we compared the receptor architecture of the dorsal bank cortex with that of areas on the ventral bank and with neighboring frontal and parietal areas and found it to be clearly distinguishable from that of adjacent areas (Fig. 11). The most prominent differences with frontal areas at the laminar level were observed in the deep layers, and this was particularly true for the 5-HT_1A_ and NMDA receptors. The 5-HT_1A_ receptor showed a bimodal pattern, with a superficial peak in layers I–II and a second, much lower peak in layers V–VI. The difference in maximum levels reached in both peaks was much larger in the case of frontal areas mainly because the deeper peak reached higher densities in the cingulate gyrus/dorsal bank of the cingulate sulcus relative to the superficial peak. That is, the superficial layers have red/orange throughout all analyzed areas. But in the deeper layers of frontal cortex, we found a predominance of green and blue colors, coding for lower densities than the yellow found in the corresponding layers of the cingulate areas. For the NMDA receptor we found a comparable situation, with clearly lower densities in the lower areas of the frontal cortex.

**Fig. 12:**
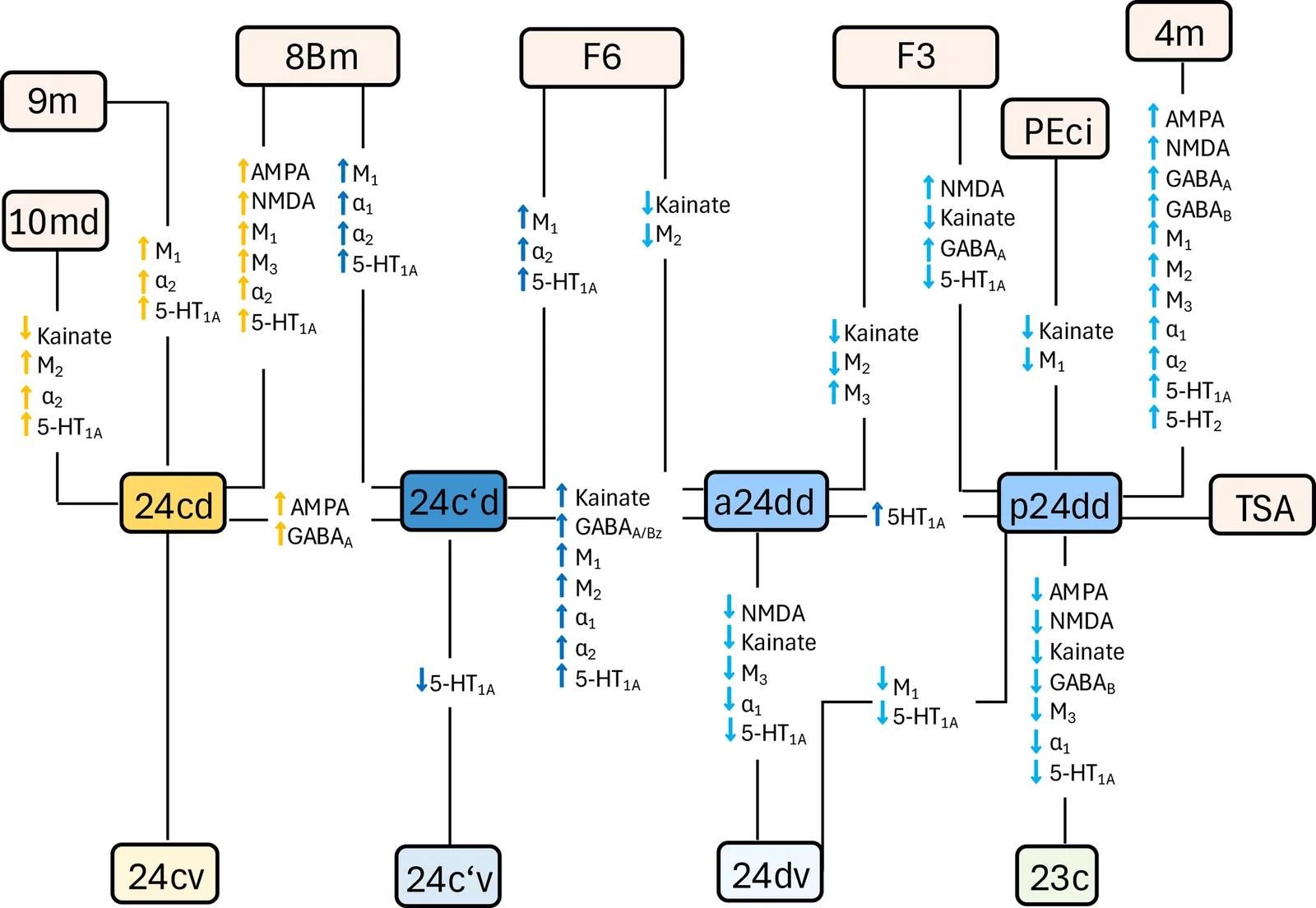
Schematic summary of the results of statistical analyses comparing receptor densities of newly identified areas 24cd (orange), 24c’d (dark blue), a24dd and p24dd (light blue) on the dorsal wall of the cingulate sulcus with the corresponding cingulate areas 24cv (transparent orange), 24c’v (transparent dark blue), 24dv (transparent light blue) and 23c (transparent green) on the ventral wall, as well as with adjacent prefrontal 10md, 9 m and 8BM, (pre)motor F6, F3 and 4 m, and parietal areas TSA and PEci, whose absolute receptor fingerprints are provided in the Fig. S7. Black lines indicate the pairs of areas subjected to statistical comparison. The direction of significant differences is represented by color-coded arrows corresponding to the dorsal bank areas: upward-right arrows denote significantly higher receptor densities relative to the dorsal bank area, whereas downward-right arrows indicate significantly lower densities. Table S1 provides the corresponding FDR-corrected p-values for all statistical comparisons

Comparisons of receptor fingerprints (Fig. 12, Fig. S7, Table S1) demonstrated that areas on the dorsal bank of the cingulate sulcus differ significantly from those of surrounding areas. Thus, they are not merely transitional cortex between cingulate, frontal, and parietal regions. Although the fingerprints of areas 24cd and 24cv, and of areas p24dd and TSA differed in shape, no single receptor reached the level of significance.

Rostral areas 24cd and 24c’d consistently showed significantly higher α_2_ and 5-HT_1A_ densities compared to their adjacent prefrontal areas 10md, 9 m and 8Bm, as well as to posterior area a24dd. Caudal areas a24dd and p24dd showed significantly lower kainate densities relative to ventral cingulate areas 24dv and 23c, respectively, and to the dorsally adjacent parietal area PEci and premotor areas F3 and F6. The primary motor area 4 m, with its uniquely small receptor fingerprint, constituted an exception. Interestingly, a24dd revealed more differences relative to its ventral counterpart 24dv than did p24dd, which only showed significantly lower M_1_ and 5-HT_1A_ densities compared to 24dv.

### Multivariate analyses

#### Intra-regional variability and deviation from the anterior–posterior gradient

**Fig. 13:**
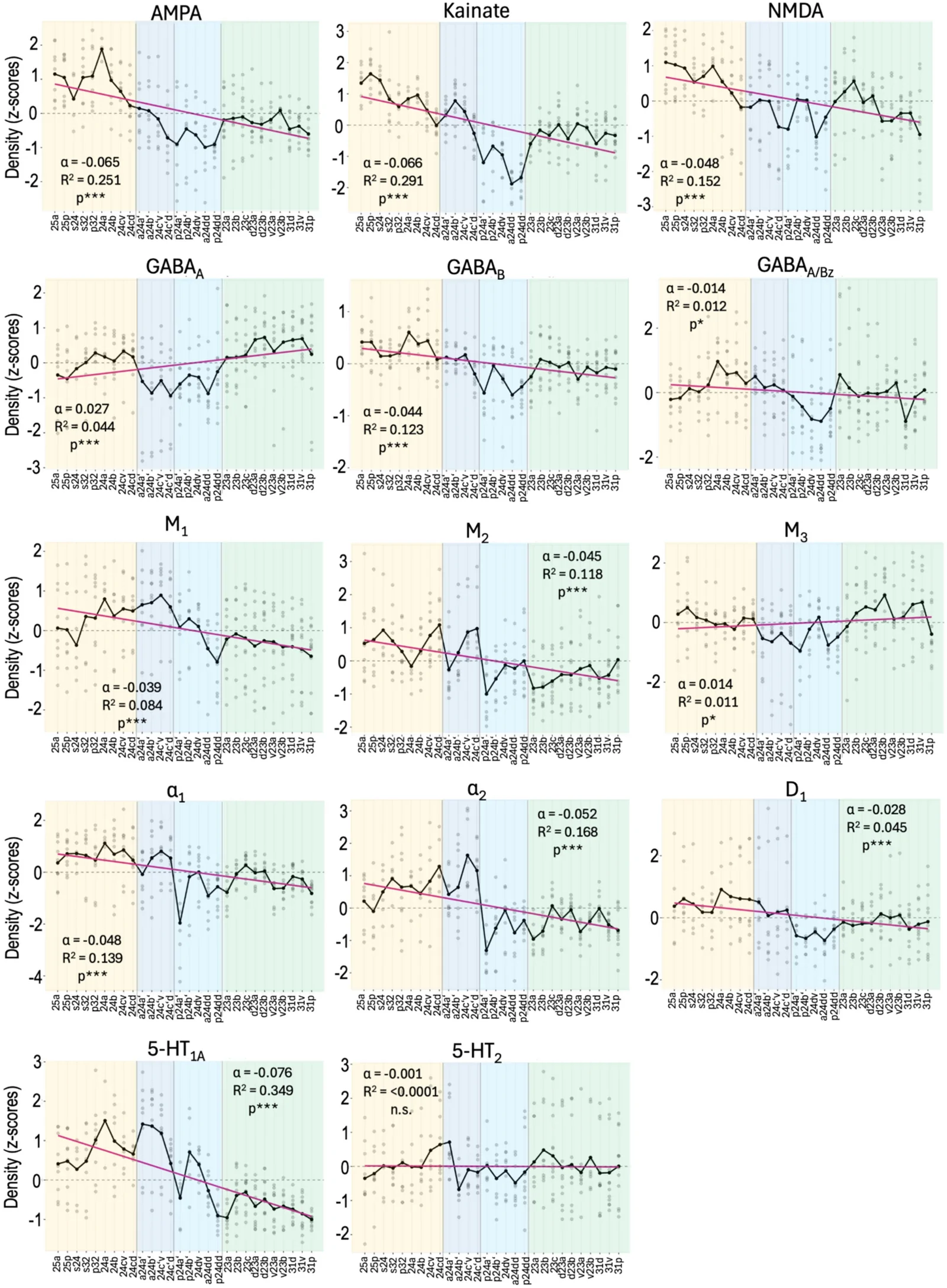
Receptor density gradients along the rostro-caudal axis of the cingulate cortex. Receptor densities (z-scores across brains) are plotted along the rostro-caudal axis. Grey dots represent individual area values, overlaid with the mean z-score (black line). The magenta line shows the linear regression fit (slope α, R^2^, p-values are reported in Table S2). Background shading indicates neurochemically defined regions: ACC (orange), aMCC (dark blue), pMCC (light blue), and PCC (green), highlighting region-specific receptor distribution patterns.

Linear regression analyses revealed systematic anterior–posterior trends in receptor densities across the cingulate cortex (Fig. 13 and Table S2). For most receptors, regression slopes indicated a decline from the anterior to the posterior cingulate regions. In contrast, GABA_A_ displayed positive slopes, with densities increasing toward posterior regions. Only the slopes for M_3_ and 5-HT_2_, and D_1_ did not reach statistical significance.

However, these trends did not reflect a continuous molecular gradient (Fig. 13). Instead, receptor densities were characterized by region-specific deviations and abrupt transitions, indicating that individual cingulate regions represent distinct molecular compartments rather than successive points along a smooth axis. Even the 5-HT_1A_ receptor, which showed the steepest overall slope, displayed a local increase in density within the aMCC compared to ACC. Interestingly, this was accompanied a sharp a sharp reduction in density at the transition from pMCC to PCC. A similar pattern, but with the sharp reduction found between aMCC and pMCC was shown by the kainate, GABA_A/Bz_, M_1_, M_2_, α_1_, and α_2_ receptors.

#### Receptor-based segregation defines four cingulate regions

**Fig. 14:**
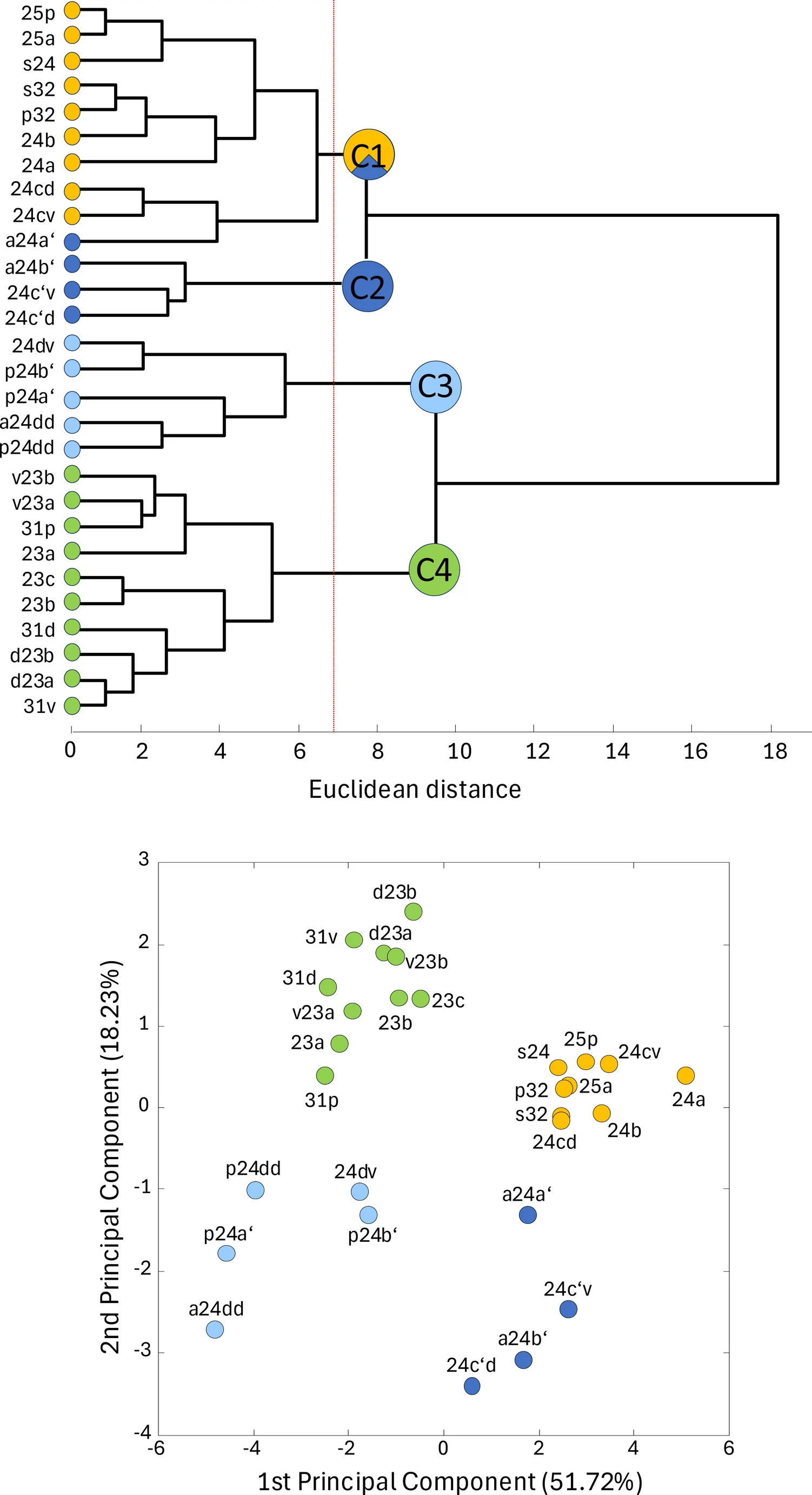
Clustering analysis of cingulate areas. Hierarchical cluster analysis (HCA; top) of 28 cingulate areas revealed four clusters (ACC areas together with aMCC area a24a′ = orange/dark blue, aMCC areas = dark blue, pMCC areas = light blue, PCC areas = green). Principal component analysis (PCA; bottom) illustrates the segregation of those areas in reduced-dimensional space, with data points color-coded according to the anatomical region to which area belongs (ACC = orange, aMCC = dark blue, pMCC = light blue, PCC = green). Multivariate analyses were based on z-score normalized receptor fingerprints (Fig. S8). K-means clustering and elbow analysis identified four as the optimal number of clusters (Fig. S9)

Hierarchical clustering and the elbow method on the within-cluster sum of squares revealed four clusters (Fig. 14; Figs. S8 and S9) corresponding closely to cytoarchitectonically defined regions. Cluster 1 (C1) included all ACC areas along with aMCC area a24a’, while cluster 2 (C2) comprised the remaining aMCC areas. Clusters 3 (C3) and 4 (C4) contained all pMCC and PCC areas, respectively. Overall, the primary division separates anterior regions (ACC/aMCC) from posterior regions (pMCC/PCC), reflecting a principal neurochemical segregation across the cingulate cortex. This was clearly reflected in the size of receptor fingerprints, since ACC and aMCC areas had the largest fingerprints, PCC areas displayed intermediate-sized ones, and pMCC areas the smallest (Fig. S8). Furthermore, ACC areas are characterized by smoother and more rounded normalized fingerprints. Specifically, ACC areas did not show strong deviations in which some receptors exhibited markedly higher-than-average densities (peaks) while others showed markedly lower-than-average densities (indentations).

Clusters C1 and C4 are the largest ones, and relatively heterogeneous in their composition. Within ACC, the subcallosal areas, the gyral areas and the sulcal areas are neurochemically most similar to each other. Indeed, sulcal areas have above average 5-HT_2_ densities (Fig. S8), which is opposite to what we see in the remaining ACC. Interestingly, subcallosal areas 25a, 25p and s24 were closely grouped together, and their fingerprints showed more peaks and indentations than the rest of the ACC areas, but not as pronounced as in MCC and PCC areas. In C4, most dysgranular subdivisions of area 23 (23b and 23c) cluster with its dorsal granular subdivisions. Interestingly, 31 d and 31v also cluster with these dorsal subdivisions of 23, whereas 31p is neurochemically more similar to the ventral subdivisions, as is dysgranular area 23a (Fig. 14).

Finally, a principal component analysis was applied to reduce the 14-dimensional receptor space to a two-dimensional representation, enabling visualization of the relative positioning of cingulate areas (Fig. 14). The first principal component primarily segregated rostral regions (ACC and aMCC) from caudal regions (pMCC and PCC). The second principal component highlights heterogeneity within these two groups, segregating the rostral regions from each other and also the posterior regions from each other.

#### Region-specific statistical differences in receptor densities

**Fig. 15:**
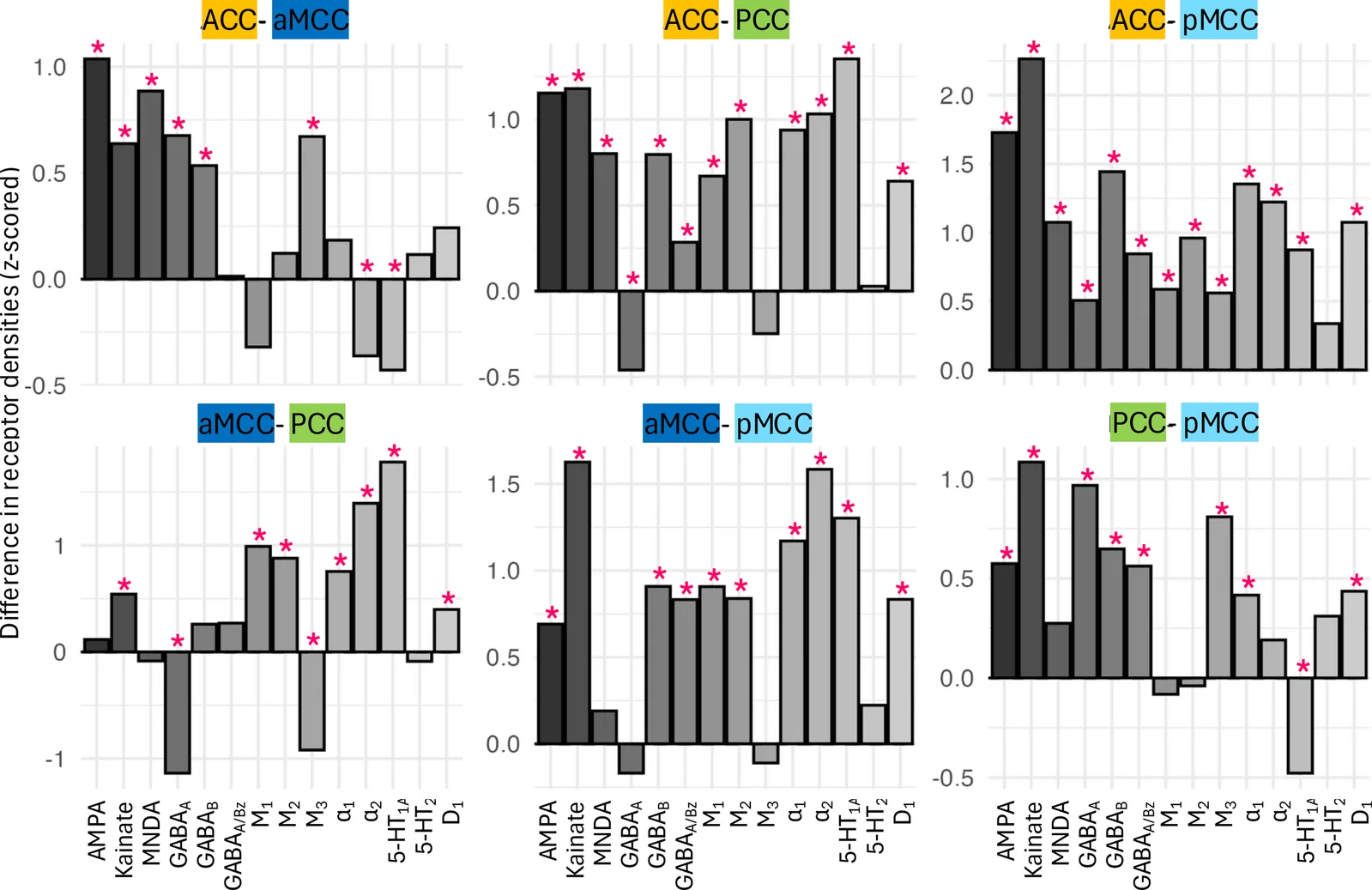
Regional differences in receptor densities across cingulate regions. Bar plots illustrate pairwise differences in receptor densities (z-scored) between the four major cingulate regions: ACC, aMCC, pMCC, and PCC. Each panel shows the difference in mean receptor density between the two regions indicated at the top of the plot (ACC–aMCC, ACC–PCC, ACC–pMCC, aMCC–PCC, aMCC–pMCC, and PCC–pMCC). The x-axis lists the 14 analyzed receptor types. Bar height represents the magnitude and direction of the difference in receptor densities (z-scores) between the compared regions. Positive values indicate higher receptor densities in the first region listed in the panel title, whereas negative values indicate higher densities in the second region. Pink asterisks mark receptor types showing statistically significant differences between the respective regions. A complementary overview of statistically significant receptor differences between the four main cingulate regions is provided in the heatmap (Fig. S10)

 To determine which receptor types contributed most to the regional differentiation, statistical comparisons were performed across cingulate regions (Fig. 15, Fig. S10). The ACC had significantly higher AMPA, kainate, NMDA, GABA_A_, GABA_B_ and M_3_ receptor densities compared to aMCC, whereas only α_2_ and 5-HT_1A_ receptors were significantly higher in aMCC relative to ACC. The ACC and aMCC exhibited significantly higher receptor densities than both the pMCC and PCC for nearly all receptor types, with the notable exception of GABA_A_, which was significantly elevated in the PCC relative to all other cingulate regions; M_3_ receptor densities were also higher in the PCC compared with the aMCC and pMCC (reaching statistical significance) and showed a similar, though non-significant, increase relative to the ACC. Notably, 5-HT_2_ was the only receptor type that did not differ significantly across any region.

## Discussion

The present findings demonstrate that, the cytoarchitectonic features enabling the definition of areas within the macaque cingulate cortex (Vogt et al. 2005) are accompanied by receptor architectonic differences which reveal a more complex organization than hitherto described. Multivariate receptor analyses consistently segregated a robust segregation of areas belonging to ACC and aMCC from those located in pMCC and PCC, thus prompting the definition of four principal cingulate regions in the macaque brain: ACC, aMCC, pMCC and PCC. This molecular segregation, together with the complementary distribution of amygdalar and inferior parietal projections to the anterior and posterior portions of the cingulate cortex, respectively (Vogt and Pandya 1987), confirms the existence of subregions within ACC and PCC, but speaks against categorizing aMCC and pMCC as subregions. Rather, they should be considered as structurally and functionally distinct regions. In the following sections, we first discuss how the here described molecular segregation of the macaque cingulate cortex into regions and subregions relates to established functional specializations (e.g., Chang et al. 2013; Rudebeck et al. 2014) and then evaluate the extension of the cingulate cortex onto the dorsal bank of the cingulate sulcus in the framework of earlier parcellation schemes (e.g., Matelli et al. 1991; Vogt et al. 2005), and the location of cingulate motor areas (Dum and Strick 2002).

### Receptor architecture and functional specialization identify four cingulate regions

While cytoarchitectonic analysis confirmed the existence of the three cingulate regions identified by Vogt et al. (2005) – namely ACC, MCC and PCC – neurochemical patterns demonstrated pronounced rostro–caudal differences within the MCC, indicating that the aMCC and pMCC should be considered as distinct regions and not mere subdivisions of MCC. This segregation is notable given that aMCC and pMCC are both agranular, as are subdivisions of ACC area 24, whereas PCC areas exhibit a well-developed granular layer IV. Thus, the observed neurochemical differentiation cannot be explained solely by classical cytoarchitectonic features, indicating a partial dissociation between laminar architecture and molecular organization (Palomero-Gallagher and Zilles, 2019). Subsequent statistical analyses demonstrated widespread differences across these four regions spanning multiple neurotransmitter systems, thus showing that this cingulate segregation is systematic.

Our molecular analyses revealed heterogeneity within the ACC cluster, distinguishing the sACC located below the genu of the corpus callosum (areas 25a, 25p, s24), whereas the pACC was further divided into gyral cortex rostral and dorsal to the genu (areas 32, 24a, 24b) and sulcal cortex within the anterior cingulate sulcus (areas 24cv, 24cd). At the functional level, different portions of the ACC have been implicated in diverse aspects of reward-guided behavior (Amiez et al. 2006), outcome monitoring and decision-making under conflict (Hayden et al. 2011; Kerns et al. 2004; Shackman et al. 2011), as well as in social valuation and social behavior (Noonan et al. 2014; Rudebeck et al. 2006; Sallet et al. 2011). The subgenual cortex is implicated in sustained motivational and affective processing (Rudebeck et al. 2014). Among the gyral areas, area 24a exhibited the highest receptor densities across several receptor types, making it one of the most neurochemically enriched subdivisions not only within the ACC but across the cingulate cortex. Interestingly, electrophysiological recordings performed by Chang et al. (2013) showed that neurons within areas 24a and 24b selectively encode reward outcomes delivered to others, whereas neurons in sulcal ACC (i.e., within 24cv and 24cd) were predominantly self-referenced. These observations suggest functional differentiation between gyral and sulcal portions of the ACC, with the gyral cortex contributing more prominently to socially oriented processing (Rudebeck et al. 2006; Sallet et al. 2011; Chang et al. 2013; Noonan et al. 2014). We found sulcal areas 24cv and 24cd to be characterized by relatively higher 5-HT_2_ receptor densities compared to rest of the ACC. Given evidence that these areas signal the value of one’s own actions and updates these representations based on feedback, this serotonergic receptor enrichment may indicate enhanced sensitivity to modulatory influences relevant for feedback-guided behavioral adjustment and approach–avoidance decision-making (Amemori and Graybiel 2012; Procyk et al. 2000).

Although aMCC is often grouped with the ACC in neuroimaging and lesion studies (Apps et al. 2016; Monosov et al. 2020; Rudebeck et al. 2006), their functional roles diverge. Both ACC and aMCC contribute to strategic exploration under uncertainty, yet ACC activity increases when information carries long-term value, whereas aMCC is more responsive to feedback availability, consistent with a role in monitoring and updating action strategies (Jahn et al. 2023). At the receptor level, the aMCC showed relatively higher densities of modulatory receptors (M_1_, α_2_ and 5-HT_1A_) and comparatively lower glutamatergic and GABAergic receptor densities than the ACC. This pattern suggests a shift in receptor balance toward stronger neuromodulatory influence relative to fast excitatory–inhibitory transmission. The aMCC receives nociceptive input through spinothalamic pathways projecting to cingulate motor regions (Dum et al. 2009; Hatanaka et al. 2003) and has been implicated in avoidance responses to noxious stimuli in macaques, suggesting a role in coordinating defensive or adaptive behavioral reactions to aversive events.

In contrast, pMCC areas were characterized by generally the lowest receptor densities throughout the cingulate cortex. This neurochemical architecture is associated with a more execution-focused circuitry resulting from extensive connectivity with (pre)motor and parietal areas (Carmichael and Price 1996; Dum and Strick 1991; He et al. 1995; Vogt and Pandya 1987), which themselves also display relatively low overall receptor densities (Niu et al. 2020; Rapan et al. 2021). Thus, a primary role of the pMCC is to support orienting of the body in space in response to sensory stimuli, integrating inputs from multiple parietal areas to guide reflexive movements and spatially directed behaviour (Vogt 2016).

The systematic differences between aMCC and pMCC across molecular, connectional, and functional domains therefore challenges the concept of them being subdivisions of MCC and supports their interpretation as distinct cingulate regions. This is in contrast with the situation described for the human brain, where areas of both aMCC and pMCC were found to be neurochemically more similar to areas of the ACC than to those of the PCC (cf. Fig. 5B in Palomero-Gallagher et al. 2009). This divergent pattern of segregation may indicate that evolutionary reorganization of cingulate receptor architecture does not follow a uniform scaling across species, but instead involves region-specific shifts in the principal axes of molecular differentiation, likely reflecting species-specific functional specializations, which will require future detailed analyses.

PCC receives its principal thalamic input from the anterior medial (AM) nucleus, with topographically organized projections to areas 23a, 23b, and 30, while additional inputs from the lateral posterior (LP) nucleus and medial pulvinar may contribute to visually evoked activity in area 23 (Vogt et al. 1987). These anatomical organization is consistent with the established role of the PCC as a core hub of the default mode network (DMN) (Buckner et al. 2008; Mantini et al. 2011), involved in self-referential cognition and memory-related processes (Leech and Sharp 2014; Pearson et al. 2011). Resting-state fMRI studies have demonstrated that the PCC is not functionally homogeneous, but that it can be subdivided into a ventral PCC (vPCC), tightly integrated within the DMN, and a dorsal PCC (dPCC), which shows broader connectivity with both DMN regions and networks supporting externally directed behavior (Margulies et al. 2009; Vincent et al. 2007). Consistent with this functional subdivision, anatomical tracer study has demonstrated distinct thalamic connectivity patterns between dorsal and ventral portions of area 23b, suggesting functional specialization within the PCC (Shibata and Yukie 2003). This functional distinction was closely mirrored in our receptor-based clustering, where PCC areas segregated into two principal groups. A larger cluster mainly comprised areas of dPCC (i.e., 23b, 23c, d23a, d23b, 31 d and 31v), corresponding to a cingulate region functionally implicated in visuospatial orientation, cognitive control, and dynamic switching between internal and external attentional states (Leech and Sharp 2014). A smaller cluster included areas 23a and 31p grouped with areas v23a and v23b of the vPCC, which has been associated with autobiographical memory and affective processing (Andrews-Hanna et al. 2014).

To our knowledge, ours is the first description of architectonic subdivisions within area 31. Interestingly, we found the most dorsal and posterior area, 31p, to be neurochemically more similar to areas of vPCC, as also happened with dysgranular area 23a. This suggests that, unlike the segregation of aMCC and pMCC regions, the PCC and ACC do not separate into molecularly defined regions, but rather consist of functionally differentiated subregions (dPCC/vPCC and subgenual/gyral/sulcal ACC, respectively) embedded within a shared molecular architecture.

### Cingulate cortex on the dorsal bank of the macaque cgs

The anatomical status of cortex along the dorsal bank of the cingulate sulcus (cgs) has long been debated. Some authors have regarded this region as part of the cingulate cortex (Matelli et al. 1991), whereas others have interpreted it as a transitional zone between the ventrally located cingulate areas and the dorsomedial frontal cortex (George Paxinos et al. 2008), or as lacking defining cingulate features altogether (Vogt et al. 2005). Functional neuroimaging studies, however, have suggested comparable midcingulate organization in humans and macaques, including dorsal bank involvement in non-human primates (Procyk et al. 2016).

On the basis of converging cytoarchitectonic and receptor-architectonic analyses, we identify four distinct cingulate areas (24cd, 24c′d, a24dd, and p24dd) extending from the fundus of the cgs onto its dorsal bank, where they border prefrontal and motor cortices. Rather than supporting the view that the dorsal bank represents a transitional cortex, our findings indicate that it contains receptor- and cytoarchitectonically definable areas that can be distinguished from adjacent frontal and motor regions, and must be categorized as belonging to the cingulate cortex. This would be in agreement with the definition of areas 24c and 24 d by Matelli et al. (1991). Hereby, their 24c corresponds to our 24c′v and 24c′d, and their 24 d encompasses our 24dv, a24dd and p24dd.

The dorsal bank of the cgs displays characteristic cingulate cytoarchitectonic features, including a prominent layer Va populated by large pyramidal neurons. In this anatomical context, the abrupt change in laminar appearance at the fundus of the cingulate sulcus, emphasized in the cytoarchitectonic analyses of Vogt (Vogt et al. 2005) and Morecraft (Morecraft et al. 2012), is also evident in our material. However, unlike these earlier studies, we did not delineate distinct fundal areas. Instead, we identify ventral and dorsal areas, with the border between them located in the vicinity of the fundus, indicating continuity of cingulate areal organization across the sulcal depth rather than the presence of a separate fundal cingulate area. Notably, Morecraft et al. (2012) acknowledged that the appearance of the fundal portion of area 24 d may be influenced by the curvature effect in the sulcal depth, highlighting the impact of sulcal geometry on cytoarchitectonic interpretation. This interpretation is consistent with the well-established observation that the cortical ribbon undergoes geometric distortion within the sulcal fundus, where deeper layers V and VI become disproportionately thinner while superficial layers I–III appear relatively expanded (Bok 1929). Thus, given the conspicuous appearance of layer V in the cingulate cortex, such a distortion is likely to highlight apparent laminar discontinuities at the fundus, creating the impression of a distinct fundal area. Furthermore, statistical analysis of receptor fingerprints demonstrated that dorsal bank areas possess a molecular architecture which is distinct from both their ventral counterparts and adjacent frontal areas, arguing against their characterization as transitional zones.

Kennerley et al. (2006) postulated that the portion of the cgs containing our ACC areas 24cv and 24cd, and our aMCC areas 24c’v and 24c’d interacts with the locus coeruleus–norepinephrine system via its strong projections to the locus coeruleus (Aston-Jones et al. 2000; Brown et al. 2004) to regulate the balance between sustained exploitation and exploratory behaviour. In this light, the significantly higher α_2_ and 5-HT_1A_ receptor densities we observed in anterior sulcal areas 24cd and 24c’d relative to the adjacent dorsomedial prefrontal cortex (10md, 9 m, 8Bm) are compatible with a stronger engagement of noradrenergic and serotonergic neuromodulatory mechanisms in this portion of the cgs. This molecular specialization also aligns with the idea that sulcal ACC areas are positioned to be particularly responsive to monoaminergic signalling relevant for adaptive behavioural regulation (Hassani et al. 2024; Jacobs and Azmitia 1992; Porrino and Goldman-Rakic 1982; Yu and Dayan 2005).

The proposed organization of the cgs at the levels of aMCC and pMCC aligns with the functional framework of cingulate motor areas (CMAs) (Dum and Strick 2002; He et al. 1995; Procyk et al. 2016), which have been subdivided into rostral (CMAr) and caudal (CMAc) – further divided into dorsal (CMAd) and ventral (CMAv) – areas based on their relative position within the cgs (He et al. 1995). These three areas also differ in their structural (Dum and Strick 1991; Hatanaka et al. 2003; He et al. 1995; Luppino et al. 1993; Morecraft et al. 2004, 2012; Petrides and Pandya 2006) and functional (Procyk et al. 2016) connectivity patterns.

aMCC areas 24c′v and 24c′d are found at a position comparable to that of CMAr, which plays a crucial role in the frontal medio-lateral interactions subserving complex adaptive decision-making (Procyk et al. 2016) particularly during feedback-driven behaviour (Quilodran et al. 2008). Interestingly, dopaminergic innervation to the cingulate cortex is densest in its anterior portions and decreases gradually towards the PCC (Berger et al. 1988, 1991; Gaspar et al. 1989; Williams and Goldman-Rakic 1998), and we found aMCC areas to have significantly higher densities of D_1_ receptors relative to pMCC areas.

Within pMCC, area 24dv corresponds to CMAv, which has been shown to contain a forelimb representation (Luppino et al. 1991). In contrast, the somatotopic organization of CMAd is more complex, because it contains a hindlimb representation anteriorly and a forelimb representation anteriorly (Luppino et al. 1991, 1993), at positions occupied by our areas a24dd and p24dd, respectively. In addition, anatomical tracing studies indicate that CMAd and CMAv differ in their corticospinal termination patterns, engaging distinct populations of spinal interneurons (Dum and Strick 1996), thereby suggesting further specialization in distal versus proximal motor control.

## Conclusion

The present findings demonstrate that cytoarchitectonic features defining areas of the macaque cingulate cortex are accompanied by distinct receptor architectures, revealing a more differentiated organization than previously described. Multivariate analyses showed that differences between the anterior and posterior midcingulate cortex were robust enough to support their classification as distinct regions, rather than subdivisions of a single MCC. In contrast, receptor architectonic variation within the ACC and PCC revealed consistent intra-regional heterogeneity, distinguishing subgenual, gyral, and sulcal subdivisions in the ACC and dorsal and ventral subdivisions in the PCC. However, these differences remained embedded within their respective anatomical regions and did not support further segregation at the regional level.

Across the cingulate cortex, pMCC areas exhibited comparatively low receptor densities, whereas ACC and aMCC were characterized by higher densities across multiple receptor systems. Importantly, cyto- and receptor architectonic analyses further demonstrate that cortex along the dorsal bank of the cingulate sulcus comprises distinct cingulate areas extending from the sulcal fundus onto the dorsal wall, supporting its classification as part of the cingulate cortex rather than a transitional or frontal region. Together, these findings provide a refined anatomical and neurochemical framework of the macaque cingulate cortex, complemented by 3D surface and volumetric maps, and clarify the organizational principles underlying regional and subregional differentiation within the cingulate cortex.

## Supplementary Information

Below is the link to the electronic supplementary material.

Supplementary file1 (DOCX 29098 KB)

## Data Availability

The complete macaque cingulate cortex receptor atlas, including receptor density maps, surface- and volume-based parcellations, and the corresponding MEBRAINS template files, is publicly available on GitHub (https://github.com/Rpn03/macaque_cingulate_receptor_maps). In addition, individual cytoarchitectonic areas are available as separate datasets through the MEBRAINS Multilevel Macaque Brain Atlas project on the EBRAINS platform (https://search.kg.ebrains.eu/instances/e39a0407-a98a-480e−9c63-4a2225ddfbe4). Each dataset includes receptor density data together with surface- and volume-based maps defining the location and extent of the corresponding area.
