# Supplementary material for: Receptor architecture shapes the regional organization of the macaque cingulate cortex
Source: Brain Struct Funct. 2026 Oct 9;231(8):148. doi: 10.1007/s00429-026-03186-6 (PMC13652784; doi:10.1007/s00429-026-03186-6)
Supplement: Supplementary file 1 [file 429_2026_3186_MOESM1_ESM.docx]

**Figure S1.** *Quantitative analysis of cytoarchitectonic borders between area 24cd and its adjacent areas 9m and 24cv. The position of each border, as determined by statistical analysis of the Mahalanobis distance, is indicated by a pink line (corresponding profile index) and marked by pink arrows on the GLI image. The corresponding dot plot (shown to the right of the GLI image) demonstrates that the locations of significant maxima in the distance function (x-axis; each indicated by a pink dot) are consistent across different block sizes (y-axis). This stability across multiple block size intervals (reflected by the vertical alignment of pink dots) confirms the reliability of the identified borders. While the prominence of individual borders varied, all were statistically significant and consistently detected across block sizes, supporting their validity. Minor deviations from observer-defined borders were observed, which likely reflect the sensitivity of the statistical approach to local cortical curvature and sulcal morphology.*

**Figure S2.** *Quantitative analysis of cytoarchitectonic borders between area 24c’d and its adjacent areas F6 and 24c’v. For details see Fig. S1.*

**Figure S3.** *Quantitative analysis of cytoarchitectonic borders between area a24dd and its adjacent areas F3 and 24dv. For details see Fig. S1.*

**Figure S4.** *Quantitative analysis of cytoarchitectonic borders between area p24dd and its adjacent areas 4m and 23c. For details see Fig. S1.*

**Figure S5.** *Volumetric representation of a macaque hemisphere showing the location and extent of cyto- and receptor architectonic areas of the cingulate cortex. The 28 identified cingulate areas are color-coded according to their absolute mean densities across the 14 receptor types analyzed. White lines indicate the borders of molecular regions (ACC, aMCC, pMCC and PCC; as shown in the volumetric distribution of the AMPA receptor). Sagittal sections at –1, –5, and –6 (medial view) illustrate their spatial distribution along the x-axis. The scale bar indicates receptor density (fmol/mg protein) for each receptor type.*

**Figure S6****.** *Laminar receptor distribution patterns. On the left, schematic drawings of coronal sections processed for receptor labeling illustrate the positions of the cingulate regions of interest (red squares) at different rostrocaudal levels: ACC (first row), aMCC (second row), pMCC (third row), and PCC (fourth row). The PCC level also includes the pMCC area p24dd because it is located at the same rostrocaudal level as the dysgranular areas 23. The respective adjacent frontal areas are shown at each level. On the right, the corresponding section profiles illustrate the laminar distribution of the remaining 10 receptor types within the regions of interest. Lines indicate the borders between the defined cingulate areas. Scale bars represent receptor densities (fmol/mg protein).*

**Figure S7.** *Absolute receptor fingerprints of the 8 frontal and parietal areas adjacent to the newly identified areas on the dorsal bank. Each radial plot shows the absolute mean densities (in fmol/mg protein) of the respective receptor types for each area. Receptor types are numbered around the plots and correspond to the legend in the right. Axes are scaled identically across all area plots.*

**Figure S8.** *Each radial plot shows z-score values of each receptor type for the respective area. In the plot, the black line represents the mean value of each receptor across all examined areas. Values below line indicate lower-than-average receptor densities for that receptor type, whereas values above indicate higher-than-average densities. Receptor types are numbered around the plots and correspond to the legend in the bottom left. Axes are scaled identically across all area plots. Areas within the dashed square represent fingerprints of subdivisions of areas 24 at the level of ACC (orange), aMCC (dark blue), pMCC (light blue), and dysgranular areas 23 at the level of PCC (green). The remaining ACC and PCC areas are shown below the square in respective colors.*

**Figure S9.** *Identification of the optimal number of clusters using the elbow method. The within-cluster sum of squares (WCSS) decreases with increasing cluster number, with an “elbow” point at k = 4, marked by a red dashed line, indicating the optimal clustering solution*.

**Figure S10.** *Heatmap illustrating statistically significant receptor differences between the four main cingulate regions based on FDR-corrected p-values (< 0.05). P-values are displayed in each square.* *White squares indicate non-significant comparisons. Red tones represent significantly higher receptor densities, whereas purple tones indicate significantly lower receptor densities. Colour intensity reflects the magnitude of the difference.*

**Table S1** *Table showing p-values for receptor differences between areas on the dorsal wall of the cingulate sulcus (24cd, 24c’d, a24dd, and p24dd) and adjacent cingulate, prefrontal, (pre)motor, and parietal areas. Statistically significant differences (FDR-corrected p < 0.05) are highlighted in yellow.*

| **Paired areas** | **AMPA** | **Kain** | **NMDA** | **GABA_A_** | **GABA_B_** | **GABA_A/Bz_** | **M_1_** | **M_2_** | **M_3_** | **α_1_** | **α_2_** | **5-HT_1A_** | **5-HT_2_** | **D_1_** |
| --- | --- | --- | --- | --- | --- | --- | --- | --- | --- | --- | --- | --- | --- | --- |
| **24cd - 10md** | 0.094 | 0.034 | 0.311 | 0.106 | 0.565 | 0.217 | 0.363 | 0.034 | 0.322 | 0.257 | 0.007 | 0.043 | 0.508 | 0.625 |
| **24cd - 9m** | 0.156 | 0.354 | 0.372 | 0.881 | 0.401 | 0.449 | 0.034 | 0.203 | 0.536 | 0.536 | 0.001 | 0.032 | 0.533 | 0.574 |
| **24cd - 8Bm** | 0.012 | 0.708 | 0.014 | 0.129 | 0.088 | 0.688 | 0.012 | 0.499 | 0.026 | 0.073 | 0.000 | 0.001 | 0.395 | 0.260 |
| **24cd - 24cv** | 0.284 | 0.236 | 0.391 | 0.772 | 0.117 | 0.555 | 0.909 | 0.499 | 0.936 | 0.354 | 0.380 | 0.753 | 0.786 | 0.975 |
| **24c'd - 8Bm** | 0.930 | 0.777 | 0.277 | 0.615 | 0.607 | 0.938 | 0.006 | 0.669 | 0.759 | 0.046 | 0.000 | 0.004 | 0.395 | 0.585 |
| **24c'd - F6** | 0.452 | 0.921 | 0.311 | 0.780 | 0.500 | 0.153 | 0.018 | 0.748 | 0.149 | 0.102 | 0.003 | 0.042 | 0.422 | 0.585 |
| **24c'd - a24dd** | 0.532 | 0.000 | 0.602 | 0.941 | 0.088 | 0.040 | 0.010 | 0.003 | 0.884 | 0.000 | 0.000 | 0.028 | 0.500 | 0.064 |
| **24c'd - 24c'v** | 0.207 | 0.078 | 0.165 | 0.424 | 0.127 | 0.758 | 0.488 | 0.839 | 0.467 | 0.532 | 0.335 | 0.019 | 0.897 | 0.932 |
| **a24dd - F6** | 0.930 | 0.000 | 0.602 | 0.719 | 0.319 | 0.643 | 0.873 | 0.011 | 0.192 | 0.038 | 0.118 | 0.890 | 0.122 | 0.259 |
| **a24dd - F3** | 0.263 | 0.002 | 0.147 | 0.066 | 0.526 | 0.822 | 0.515 | 0.005 | 0.024 | 0.064 | 0.137 | 0.900 | 0.424 | 0.074 |
| **a24dd - p24dd** | 0.858 | 0.699 | 0.220 | 0.143 | 0.500 | 0.456 | 0.433 | 0.584 | 0.495 | 0.377 | 0.405 | 0.034 | 0.500 | 0.574 |
| **a24dd - 24dv** | 0.431 | 0.039 | 0.020 | 0.308 | 0.253 | 0.929 | 0.208 | 0.848 | 0.028 | 0.022 | 0.119 | 0.032 | 0.459 | 0.627 |
| **p24dd - F3** | 0.129 | 0.006 | 0.004 | 0.000 | 0.942 | 0.614 | 0.909 | 0.022 | 0.002 | 0.339 | 0.580 | 0.044 | 0.897 | 0.332 |
| **p24dd - 4m** | 0.046 | 0.048 | 0.001 | 0.000 | 0.000 | 0.371 | 0.018 | 0.000 | 0.000 | 0.002 | 0.000 | 0.027 | 0.001 | 0.088 |
| **p24dd - TSA** | 0.877 | 0.081 | 0.363 | 0.119 | 0.259 | 0.555 | 0.127 | 0.460 | 0.094 | 0.271 | 0.317 | 0.325 | 0.439 | 0.627 |
| **p24dd - PEci** | 0.825 | 0.017 | 0.382 | 0.155 | 0.066 | 0.950 | 0.016 | 0.492 | 0.360 | 0.524 | 0.927 | 0.585 | 0.634 | 0.574 |
| **p24dd - 23c** | 0.018 | 0.000 | 0.017 | 0.282 | 0.024 | 0.456 | 0.078 | 0.114 | 0.015 | 0.018 | 0.285 | 0.034 | 0.260 | 0.761 |
| **p24dd - 24dv** | 0.514 | 0.075 | 0.319 | 0.719 | 0.607 | 0.532 | 0.021 | 0.754 | 0.141 | 0.138 | 0.508 | 0.000 | 0.948 | 0.899 |

**Table S2.** *Linear regression slopes (α) across cingulate regions with corresponding confidence intervals (lower and upper bounds), coefficients of determination (R²), and FDR-corrected p-values (p < 0.05).*

| **Receptor** | **α** | **α_lower** | **α_upper** | **R^2^** | **p-value** |
| --- | --- | --- | --- | --- | --- |
| **AMPA** | -0.0649963 | -0.0767209 | -0.0532718 | 0.25133479 | 1.32E-21 |
| **Kainate** | -0.0658318 | -0.0781187 | -0.0535449 | 0.29070692 | 5.17E-21 |
| **NMDA** | -0.0480019 | -0.0613874 | -0.0346164 | 0.15249172 | 3.10E-11 |
| **GABA_A_** | 0.02737182 | 0.01525148 | 0.03949215 | 0.04416387 | 1.99E-05 |
| **GABA_B_** | -0.0435187 | -0.0573339 | -0.0297035 | 0.12333341 | 4.10E-09 |
| **GABA_A/Bz_** | -0.0144333 | -0.0271514 | -0.0017152 | 0.01244982 | 0.03142765 |
| **M_1_** | -0.0394945 | -0.0474444 | -0.0315447 | 0.08447934 | 1.14E-18 |
| **M_2_** | -0.0446768 | -0.0558861 | -0.0334674 | 0.11834529 | 2.37E-13 |
| **M_3_** | 0.01354015 | 0.00034571 | 0.02673459 | 0.0111776 | 0.04877304 |
| **α_1_** | -0.0475956 | -0.0593729 | -0.0358183 | 0.13960303 | 1.52E-13 |
| **α_2_** | -0.0523481 | -0.0652368 | -0.0394594 | 0.16797046 | 1.52E-13 |
| **5-HT_1A_** | -0.0754548 | -0.0849389 | -0.0659707 | 0.34922154 | 7.52E-38 |
| **5-HT_2_** | 0.00052572 | -0.0089044 | 0.00995582 | 1.50E-05 | 0.91307495 |
| **D_1_** | -0.0275206 | -0.0415176 | -0.0135237 | 0.045448 | 0.00019011 |
